# Peripheral inflammation triggering central anxiety through the hippocampal glutamate metabolized receptor 1

**DOI:** 10.1111/cns.14723

**Published:** 2024-04-26

**Authors:** Jun‐Meng Wang, Yue‐Mei Wang, Yuan‐Bing Zhu, Chan Cui, Tong Feng, Qin Huang, Shu‐Qing Liu, Qiao‐Feng Wu

**Affiliations:** ^1^ Acupuncture and Moxibustion School Chengdu University of Traditional Chinese Medicine Chengdu China; ^2^ Institute of Acupuncture and Homeostasis Regulation Chengdu University of Traditional Chinese Medicine Chengdu China; ^3^ Key Laboratory of Acupuncture for Senile Disease (Chengdu University of TCM), Ministry of Education Chengdu China

**Keywords:** anxiety‐like behavior, colitis, GRM1, spatial transcriptome

## Abstract

**Aims:**

This study aimed to investigate the relationship between ulcerative colitis (UC) and anxiety and explore its central mechanisms using colitis mice.

**Methods:**

Anxiety‐like behavior was assessed in mice induced by 3% dextran sodium sulfate (DSS) using the elevated plus maze and open‐field test. The spatial transcriptome of the hippocampus was analyzed to assess the distribution of excitatory and inhibitory synapses, and Toll‐like receptor 4 (TLR4) inhibitor TAK‐242 (10 mg/kg) and AAV virus interference were used to examine the role of peripheral inflammation and central molecules such as Glutamate Receptor Metabotropic 1 (GRM1) in mediating anxiety behavior in colitis mice.

**Results:**

DSS‐induced colitis increased anxiety‐like behaviors, which was reduced by TAK‐242. Spatial transcriptome analysis of the hippocampus showed an excitatory‐inhibitory imbalance mediated by glutamatergic synapses, and GRM1 in hippocampus was identified as a critical mediator of anxiety behavior in colitis mice via differential gene screening and AAV virus interference.

**Conclusion:**

Our work suggests that the hippocampus plays an important role in brain anxiety caused by peripheral inflammation, and over‐excitation of hippocampal glutamate synapses by GRM1 activation induces anxiety‐like behavior in colitis mice. These findings provide new insights into the central mechanisms underlying anxiety in UC and may contribute to the development of novel therapeutic strategies for UC‐associated anxiety.

## Graphical abstract

Anxious behavior in mice with colitis was associated with an imbalance in hippocampal excitatory inhibition due to the overactivation of glutamate synapses. Peripheral suppression of colon inflammation or downregulation of GRM1 expression by AAV virus interference can significantly improve anxiety‐like behavior.

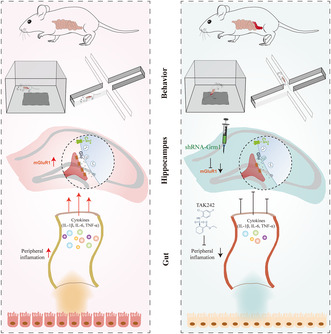

## 1 INTRODUCTION

Many gastrointestinal diseases and disorders, such as ulcerative colitis (UC), irritable bowel syndromes (IBS), and so on, are associated with depression, anxiety, and cognitive dysfunction.^1^ For instance, a growing body of research shows that anxiety is one of the most prevalent psychiatric symptoms in UC patients.^2^ The latest meta‐analysis shows that the prevalence of anxiety in UC patients is 32.6%,^3^ and more than half of patients with active UC met the criteria for anxiety symptoms.^4^ On the other hand, anxiety increases susceptibility and risk of recurrence in gastrointestinal disease patients.^5^, ^6^, ^7^ A large sample of clinical evidence shows that disease activity in UC is highly correlated with the severity of depression and anxiety.^8^ More and more studies have shown that UC is a brain–gut‐related disease.^9^, ^10^, ^11^ Changes in gut inflammation, microbiome, etc., interact with the central nervous system through the immune system and circulating metabolites and are involved in developing disorders such as anxiety, cognitive impairment, etc., but the mechanisms are still unclear.^12^, ^13^ Therefore, a comprehensive understanding of the mechanism of UC‐related anxiety and the role of gut–brain bidirectional communication in it has important clinical significance for treating gastrointestinal‐related complications and improving the life quality of patients.

As an important part of the limbic system, the hippocampus is essential in responding to systemic inflammation and regulating cognitive functions and mood, such as anxiety and depression.^14^, ^15^, ^16^ A large amount of data from different peripheral inflammatory models demonstrate the causal role of rising levels of hippocampal inflammation, neurogenesis inhibition, excitability, and inhibitory imbalance in inducing anxiety‐related symptoms.^17^, ^18^, ^19^ However, aspects of the molecular cascade linking peripheral inflammation, hippocampal dysfunction, and anxiety‐like behavior in colitis still need to be clarified, particularly which pathway or molecule directly mediates the occurrence of anxiety behavior in mice with colitis. A detailed understanding of these pathways may provide vital information for developing new strategies to combat UC comorbidities.

In this study, we observed evident anxiety‐like behavior in dextran sulfate sodium (DSS)‐induced colitis mice. The peripheral inflammation inhibitor, TAK‐242, significantly ameliorated intestinal inflammation and behavioral manifestations in DSS‐treated mice. This confirms that intestinal inflammation serves as a crucial signal in inducing anxiety. Furthermore, using spatial transcriptome analysis, we found that anxious behavior in mice with colitis was associated with an imbalance in hippocampal excitatory inhibition due to the overactivation of glutamate synapses. Finally, we demonstrate that glutamate‐metabolized receptor GRM1 is directly involved in inflammation‐induced anxiety processes and that peripheral suppression of colon inflammation or downregulation of GRM1 expression by AAV virus interference can significantly improve anxiety‐like behavior.

## 2 MATERIALS AND METHODS

### 2.1 Mice

Male C57BL/6J mice (~25 g) were obtained from Gempharmatech (Chengdu, China). Animals were fed ad libitum with standard rodent chow and water, under conditions of ambient temperature (23 ± 1°C) and a 12 h light/dark cycle in the specific pathogen‐free housing. Mice were randomized into Control group and DSS group. The protocol was approved by the Committee on the Ethics of Animal Experiments of Chengdu University of Traditional Chinese medicine.

### 2.2 Induction of colitis model

Mice in DSS group was provided with 3% dextran sodium sulfate (DSS) (MP Biomedicals) in the drinking water for 7 days, and the Control group was only administered normal drinking water.

### 2.3 TAK‐242 treatment

TAK‐242 (S7455, Selleck) was dissolved in a solution containing 5% DMSO/40% Polyethylene Glycol 300 (PEG300)/5%Tween80. Mice were intraperitoneally injected with 5 mg or 10 mg per kg of TAK‐242 solution or an equivalent volume of saline. At the higher dose used, TAK‐242 can efficiently inhibit TLR4‐mediated production of various cytokines such as TNF‐α, IL‐1β, and IL‐6.^20^

### 2.4 Disease activity index (DAI)

As we published previously^21^, DAI was calculated as a combined score of Body weight loss, Feces status, and Occult. Each subscale score is presented in Table S1.

### 2.5 Intestinal permeability assay to FITC‐dextran

FITC‐dextran permeability test was used to assess intestinal barrier integrity of mice.^22^ The experiment was carried out on the last day of exposure to DSS. Before the start of the experiment, the mice fasted for 4 h and were allowed to drink freely. FITC‐dextran was orally administered to mice at a weight of 600 mg/kg, and then the mice were returned to the cage, without food but drinking water. After 2 h of oral administration of FITC‐dextran, the mice were anesthetized and blood was collected into heparinized, light‐protected tubes, and centrifuged (10 min, 12,000 *g*, 4°C). The concentration of FITC‐dextran was analyzed using a fluorescence spectrophotometer (TECAN, Infinite M200), and I‐Control 2.0 software at the excitation wavelength of 485 nm and the emission wavelength of 528 nm.

### 2.6 Hematoxylin–eosin (HE) staining and immunofluorescence (IF)

Colon or brain tissues from the mouse model were fixed in 4% paraformaldehyde and embedded in paraffin. After deparaffinization, the sections were incubated with 10 mM sodium citrate buffer (pH 6.0) and 3% H_2_O_2_ to repress the endogenous peroxidase activity, followed by 5% BSA and 0.05% Triton X‐100 blocking treatment.

For colon HE staining, the sections were stained with HE, and the images were captured by an inverted microscope. For IF assay, primary antibodies used were anti‐PSD95 (1:50, A6194, ABclonal), anti‐GRM1 (1:50, #ab182277, Abcam). Cell nuclei were visualized with DAPI (G1012, Servicebio) and fluorescence was collected by a fluorescence microscope (3D‐Histech).

### 2.7 Behavioral tests

All behavioral tests were performed from 10 a.m to 6 p.m. The mice were transported to the behavioral room 1 h before testing. The behavioral equipment was cleaned with 75% ethanol after each test. Behavioral testing was conducted 24 h after the cessation of DSS administration or 48 h after the last TAK‐242 injection.

#### 2.7.1 Open field test

The mice were placed at the center of the test box (50 cm × 50 cm × 50 cm) at the beginning of the test and allowed to freely explore the box for 10 min. The central area of the box which accounts for 25% of the box was defined as the central arena. The behavioral test was video recorded and analyzed using automated behavioral tracking software (EthoVision, Noldus).

#### 2.7.2 Elevated plus maze

The maze was placed in the middle of the room with each arm pointing to the corner of the room. At the beginning of the test, a mouse was placed at the junction of the four arms and faced an open arm. The mouse was left in the maze for 10 min and video recorded. The duration and walking distance in each arm were analyzed using an automated behavioral tracking software (EthoVision, Noldus).

### 2.8 Spatial transcriptomics

Spatial resolved transcriptomic data was acquired using the Spatial transcriptomics kit (10x Genomics). Tissue Optimization and Library preparation were carried out according to the manufacturers protocol, as described below.

#### 2.8.1 Tissue collection and RNA quality control

The brains from two mice from Control and DSS group were included in this study. Fresh tissue collected immediately post‐resection was rapidly embedded in optimal cutting temperature compound (OCT, Sakura) which snap‐frozen by drikold. The prepared tissue samples were stored at −80°C until further processing. The samples were cryosectioned coronally at a thickness of 10 μm by Cryostat Microtome (Leica CM3050, Germany). Then 5–10 sections were selected to be stained with HE, photographed, and tested for RNA integrity number (RIN) value.

#### 2.8.2 Permeabilization, library preparation, sequencing, and data pre‐processing

The spatial transcriptomics libraries were prepared using the 10× Genomics Chromium system. Samples permeabilization was referred to Visium spatial tissue optimization kit (1,000,184, 10x Genomics). After tissue permeabilization and cDNA synthesis, library preparation, and high‐throughput sequencing, a total of 5731 spots and about 6.3 × 10^8^ reads were targeted for capture from two mice samples. The raw data were processed using the analysis pipeline Space Ranger (1.0.0) for sample demultiplexing, barcode processing, reads alignment to mouse reference genome (mm10), and gene counting.

#### 2.8.3 Spatial transcriptomics data analysis

Space Ranger (v1.0) is an official software package for Visium spatial transcriptome data analysis provided by 10x genomics. Space Ranger uses the raw data fastq files obtained by sequencing and H&E staining images of tissue sections as input data, and performs reference genome alignment, tissue detection, benchmark detection, and barcode/UMI statistics to generate spot‐gene expression matrix.

After data processing by Space Ranger, we used the seurat package (v4.0) to perform the following analysis: (1) data quality control to remove low‐quality spots; (2) normalization to eliminate capture size preference; (3) feature selection to screen for large differences in expression (4) principal component analysis to reduce the dimension of the data; (5) t‐SNE and UMAP algorithms to display the classification results in dimension reduction; and (6) “FindIntegrationAnchors” and “IntegrateData” to integrate multi‐samples.

After processing in Seurat packages, we used Loupe Browser (5.0.0) to visualize clusters and select hippocampus spots precisely according to HE staining. The edgeR package (version 3.26.8) was used to obtain differentially expressed genes of hippocampus between two groups. Gene set enrichment analysis (GSEA) was performed by clusterProfiler (4.2.0). Geneset variation analysis (GSVA) and spatial visualization of glutamate synaptic signal were done by the GSVA (1.42.0) and seurat, respectively.

### 2.9 Surgery and viral injections

Grm1 shRNA (shGrm1 group) and scrambled shRNA (NC group) were purchased from Hanheng Biotechnology (Hanheng Biotechnology). Mice were anesthetized with 5% isoflurane and kept under 2% isoflurane throughout surgery. The hair was shaved, and local sterilization was administrated. The scalp was incised to expose the skull. After removing the periosteum tissue, bilateral hippocampus (AP: −2 mm; ML: ±1.5 mm; DV: −1.5 mm) was located by the stereotaxic instrument. The injection hole was created using a high‐speed micro‐drill, and 1 μL of virus was injected into the target region using a gas‐tight Hamilton Syringe (26‐gauge, flat‐tip needle) mounted on a stereotaxic device at a rate of 0.1 μL/min. After injection, the needle was left at the injection site for 5 min before withdrawal. Mice body temperature was kept at 36°C by a heating pad during surgery. After surgery, animals were allowed to recover for 3 weeks prior to the performance of all subsequent experiments.

### 2.10 Statistical analysis

All datasets were first tested for the normality before enrolling into statistical analysis. Those fitted normal distribution were analyzed by parametric approaches. Student's *t* tests were applied for comparisons between two groups, and one‐way ANOVA analyses were applied for comparisons between multiple groups, followed by Tukey's post hoc comparison between 2 specified groups. Repeatedly measured data were analyzed by repeated measurement analysis of variance. When dataset did not pass the normality test, nonparametric approaches were employed, including Mann–Whitney comparison between 2 groups, and Kruskal–Wallis test for multi‐group comparison, in conjunction with Dunn's post hoc comparison. All statistical analyses were two‐sided, and *p* < 0.05 was considered to be significant.

## 3 RESULTS

### 3.1 DSS‐induced colitis increased anxiety‐like behaviors in mice

Consistent with our previous report^21^and all other experimental cohorts, DSS‐induced colitis mice exhibited decreased body weight (Figure S1A), reduced survival (Figure S1B), and higher DAI scores (Figure S1C). At the same time, data on colonic phenotypes (i.e., colon length, blood FITC concentration, and bloody stools; Figure S1D–H) demonstrated hyperemia, edema, and impaired barrier integrity in colonic tissue of DSS mice. Mice administered DSS also show an increase in colonic inflammation by histopathological examination (Figure S1I).

**FIGURE 1:**
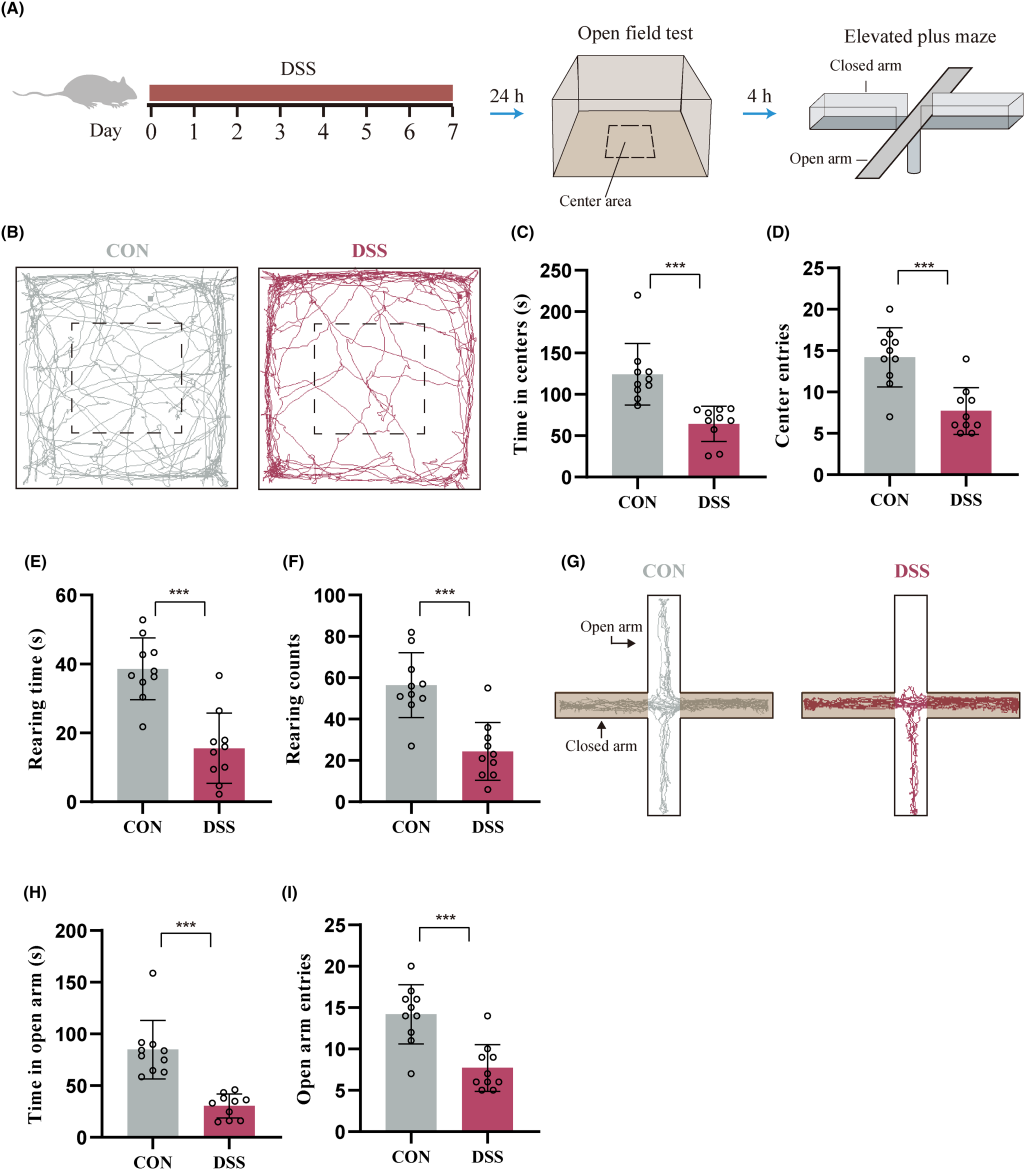
DSS‐treatment increases anxiety‐like behavior in mice. (A) Schematic diagram of the experimental procedure. Anxiety‐like behavior was accessed by open field test (OFT) and elevated plus maze (EPM) test 24 h after 7d DSS treatment (*n* = 10). (B) Representative activity tracking of 10 min in the OFT. (C) Statistical results indicate DSS‐treatment decreased the time spent in the center area during OFT. (D) DSS‐treatment decreased center entries during OFT. (E) DSS‐treatment decreased the raring time during OFT. (F) DSS‐treatment decreased the raring frequency OFT. (G) Representative activity tracking of 10 min in the EPM. (H) Statistical results indicate that DSS‐treatment decreased time spent in open arms during EPM. (I) DSS‐treatment decreased entries to open arm during EPM. ***p < 0.001.

We then measured the anxiogenic effects by using open‐field test (OFT) and elevated plus maze (EPM) (Figure 1A). DSS‐induced colitis mice spent less time in (Figure 1B,C) and had fewer entries to (Figure 1D) the center area of the OFT. The rearing time (Figure 1E) and frequency (Figure 1F) of DSS‐induced colitis mice were also decreased compared with Control mice. Results of EPM showed that the DSS‐induced colitis mice spent less time in (Figure 1G,H) and had fewer entries to (Figure 1I) the open arms. These results indicate that DSS‐treatment significantly increases anxiety‐like behavior in mice.

### 3.2 Inhibition of colon inflammation improved anxiety‐like behaviors in mice

**FIGURE 2:**
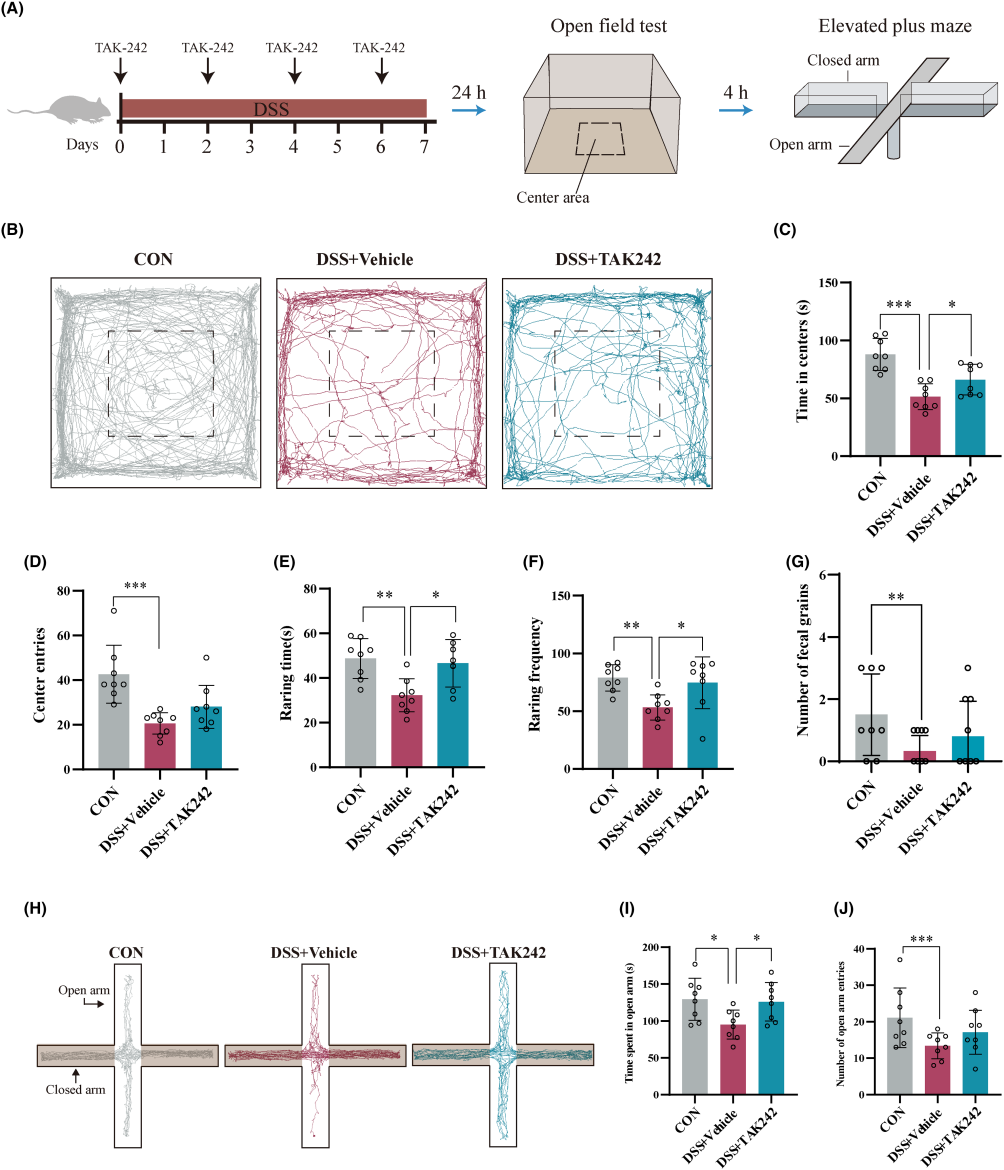
TAK‐242 improved anxiety‐like behaviors of DSS‐induced mice. (A) Schematic diagram of the experimental procedure (*n* = 8). (B) Representative activity tracking of 10 min in the OFT. (C) Statistical results indicate TAK‐242 increased the time spent in the center area during OFT. (D) TAK‐242 had no obvious effect on center entries during OFT. (E) TAK‐242 increased the raring time during OFT. (F) TAK‐242 increased the raring frequency during OFT. (G) TAK‐242 had no effect on number of fecal grains during OFT. (H) Representative activity tracking of 10 min in the EPM. (I) Statistical results indicate that TAK‐242 increased time spent in open arms during EPM. (J) TAK‐242 had no obvious effect on entries to open arm during EPM. *p < 0.05, **p < 0.01, ***p < 0.001.

Then, we used TAK‐242 (Figure S2A) into colitis mice by intraperitoneal injection, a selective inhibitor of TLR‐4, to observe whether targeted inhibition of peripheral inflammation could improve anxiety‐like behaviors in colitis mice. Compared with the DSS + Vehicle group, different doses of TAK‐242 (5 and 10 mg/kg, intraperitoneal administration) significantly alleviated the shortening of colon length and weight loss in mice with colitis and improved the DAI score (Figure S2B–E). Histological evaluation of colon tissue revealed that DSS‐induced colitis is characterized by epithelial cell destruction, crypt deformation, and inflammatory cell infiltration. After treatment with different doses of TAK‐242, the degree of colonic mucosal lesions was significantly improved, the infiltration of inflammatory cells in the mucosa and submucosa was reduced, and the integrity of the colonic mucosa was maintained (Figure S2F). In addition, compared with the 5 mg/kg group, the 10 mg/kg group had a more substantial improvement effect on colitis. More importantly, in comparison with DSS + Vehicle mice, TAK‐242 significantly alleviated DSS‐induced colitis mice anxiety‐like behavior, as reflected by increased time spent in the center region (Figure 2A–D), raring time (Figure 2E), and frequency (Figure 2F) in OFT and increased time in the open arms in the EPM (Figure 2H–J).

### 3.3 Spatial transcriptome of hippocampus

To clarify changes in the hippocampus in DSS‐induced colitis mice, we first used spatial transcriptome techniques (ST) to establish the brain, especially hippocampus transcriptome spatially. From two ST datasets of Control and DSS brain tissues, we obtained a total of 5731 ST spots (Control, 2994; DSS, 2737) covering the tissue samples, which corresponded to a total of 18 ST clusters. Figure S3A shows the spots and clusters corresponding to the neuroanatomical distinct regions of each sample, and feature similarity is encoded by colors. The result of unsupervised clustering was also plotted by the uniform manifold approximation and projection (UMAP) algorithm (Figure S3B). Figure S3C,D shows separately the individual samples in the UMAP space and the clusters proportion in each sample. The quality control measures (read count, feature count, ratio of mitochondrial genes; Figure S3E–G) confirm that there is no sequencing bias between the individual samples. The top 5 marker genes that are most highly expressed in the individual clusters are shown in Figure S4.

**FIGURE 3:**
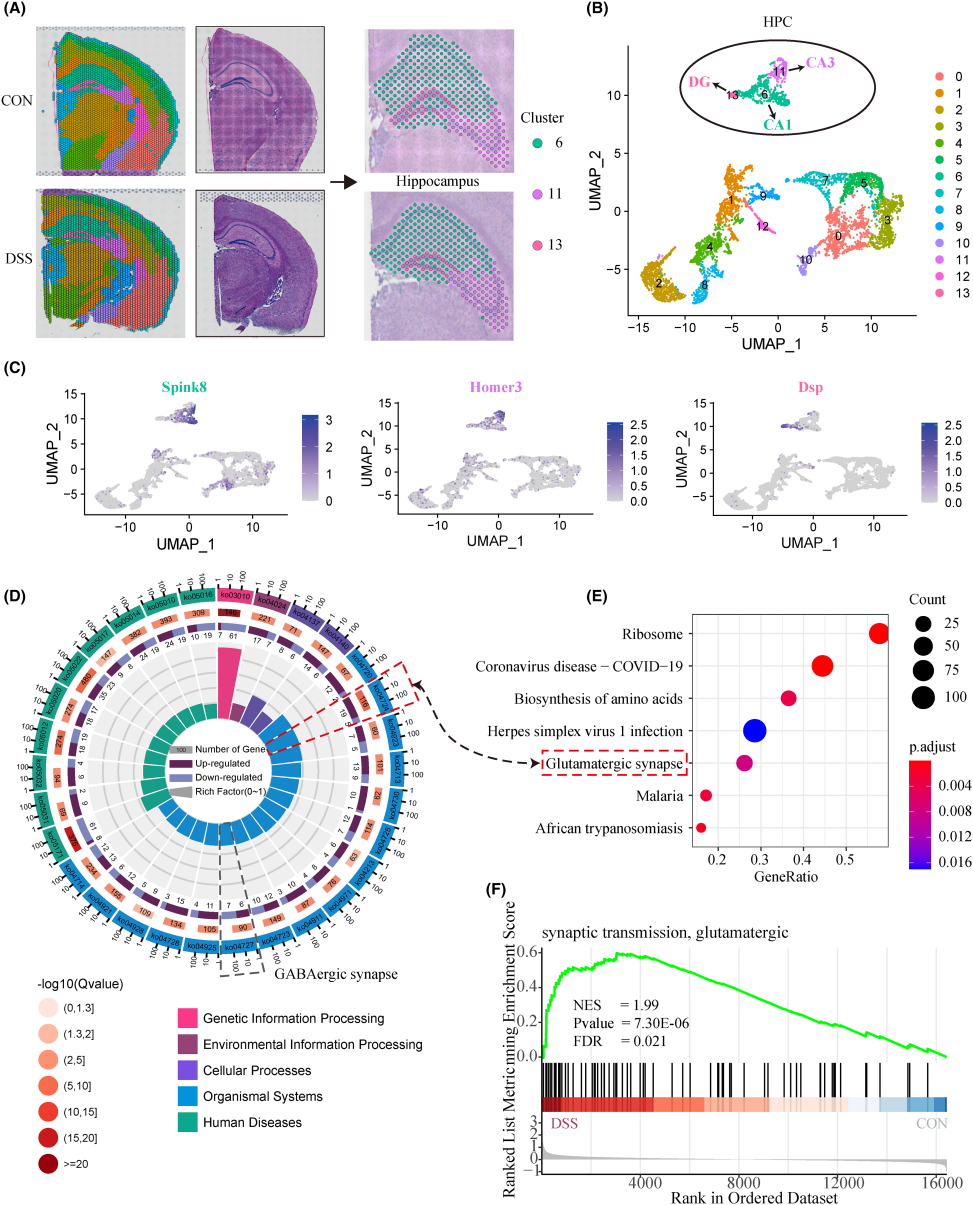
Spatial transcriptome analysis uncovers aberrant activation of hippocampal glutamate synaptic signaling in colitis mice. (A). Schematic representation of hippocampal cluster definition based on spatial anatomical structure and UMAP dimensionality reduction. Cluster 6, 11, and 13 was defined as the hippocampus. (B) Distribution of hippocampus clusters and spots of two groups in the UMAP space. (C) The distribution of verifiable markers in UMAP space demonstrates the accuracy of hippocampal clustering. (D) The circle diagram shows the KEGG enrichment analysis results of the differential genes in hippocampal. From outside to inside, the first circle represents the enrichment classification, and the scale outside the circle represents the number of genes. Different colors represent different classifications; the second circle represents the number and *Q* value of the classification in the background genes. The more genes, the longer the bar, and the smaller the value, the redder the color; the third circle represents the proportion of genes; dark purple represents the proportion of up‐regulated genes, and light purple represents the proportion of down‐regulated genes; the lower part shows the specific value; the fourth circle represents the proportion of each The RichFactor value of the term (the number of foreground genes in this category divided by the number of background genes). Each cell of the background auxiliary line represents 0.1. (E) GSEA pathway enrichment of differentially genes. The bubble size represents the number of enriched acetylated genes, and the bubble color represents the p value of each term. (F) GSEA enrichment plots indicated that glutamatergic synaptic pathway was significantly enriched in DSS‐induced colitis mice. Each group consists of one mouse brain tissue slice, with 381 (CON), 297 (DSS) spots in the hippocampal region, respectively.

Based on the correspondence between clusters and brain anatomy, we can easily obtain the spots of the hippocampus for each sample. As shown in Figure 3A, we collectively defined cluster 6, 11, and 13 as the hippocampus, which correspond to CA1, CA3, and DG subregions. Figure 3B shows the distribution landscape of hippocampal tissue in the UMAP space. To verify the accuracy of our brain region division, we aligned the marker genes of the three clusters with a spatial transcriptome‐based Molecular atlas of the adult mouse brain (MAMB).^23^ Consistent with our results (Figure 3C, Figure S5), the MAMB (Figure S6) showed that Wipf3, Hpca, and Ddn were highly expressed in the entire hippocampus, while the marker genes of cluster6 (Itpka, Fibcd1, Spink8), 11 (Cabp7, Homer3, Hs3st4), and 13 (C1ql2, Fam163b, Dsp) were specific in CA1, CA3, and DG regions, respectively. These results demonstrate that we have established robust spatial gene expression profiles of the hippocampus that can be used for subsequent differential analysis.

### 3.4 Hyperactivated glutamatergic synapse in the hippocampus is related to anxiety‐like behaviors in DSS‐induced colitis mice

After defining the hippocampal region precisely, edgeR (3.36) was performed to identify the differential expression profile of hippocampal tissue at spot resolution. A total of 235 differential genes were identified in the hippocampus of the two groups based on the criteria of **|**fold change (FC)**|**greater than 1.25 and adjusted *p* value (*Padj*) < 0.05. Compared to the Control group, the results showed that 212 genes were up‐regulated, and 23 genes were down‐regulated in DSS group (Figure S7A). Next, GO, KEGG, and GSEA functional enrichment were carried out to characterize the role of differential genes in colitis. The top GO terms showed that the differentially genes were markedly involved in the membrane depolarization, and synaptic transmission, glutamatergic (Figure S7B,C). KEGG (Figure 3D) and GSEA (Figure 3E,F) pathway enrichment analysis found that up‐regulated genes mainly enriched in the Glutamatergic synapse.

**FIGURE 4:**
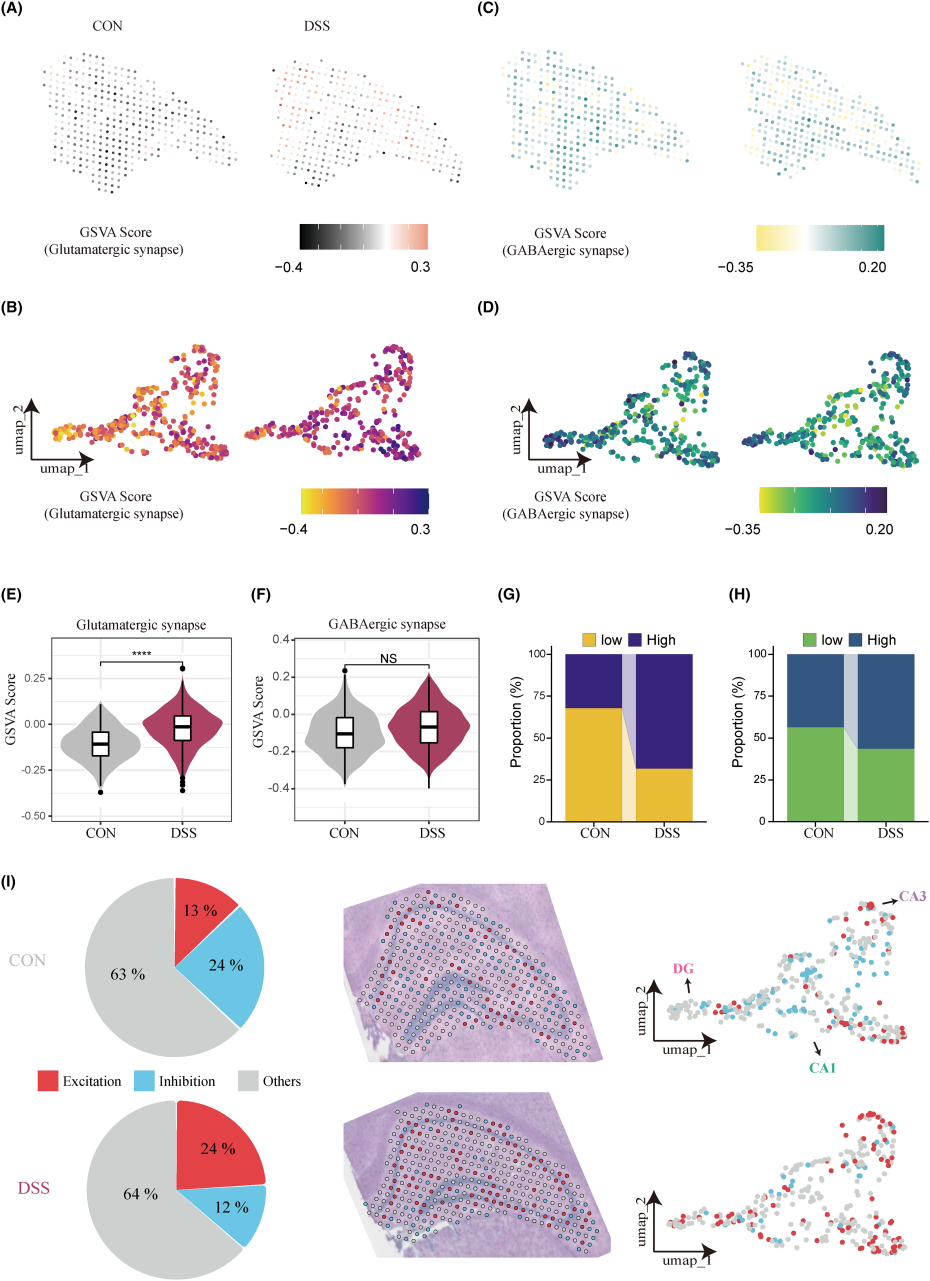
Excitatory/inhibitory imbalance in the hippocampus is mediated by abnormal activation of glutamate synaptic signaling. (A) Distribution of hippocampal glutamate synaptic signals in anatomical space based on GSVA scores. (B) Distribution of hippocampal glutamate synaptic signals in UMAP space based on GSVA scores. (C) Distribution of hippocampal GABAergic synaptic signals in anatomical space based on GSVA scores. (D) Distribution of hippocampal GABAergic synaptic signals in UMAP space based on GSVA scores. (E) Hippocampal glutamatergic synapse scores are significantly elevated in colitis mice. ****p < 0.0001. (F) Hippocampal GABAergic synapse scores did not differ significantly between groups. (G) and (H) respectively represent the proportions of different levels of glutamatergic (G) and GABAergic (H) spots in the hippocampus among the groups. (I) Proportion and distribution of excitatory and inhibitory spots defined by glutamatergic and GABAergic scores in the hippocampus of the two groups of mice. Each group consists of one mouse brain tissue slice, with 381 (CON), 297 (DSS) spots in the hippocampal region, respectively. ***p < 0.001, NS, no significance.

Given that excitatory/inhibitory imbalance in central synapses has been shown to induce anxiety‐like behaviors,^24^ we assessed Glutamatergic and GABAergic synapse scores for each spot in hippocampal tissue by GSVA analysis. The results showed that glutamatergic synaptic signaling across the hippocampus was significantly activated in the DSS group (Figure 4A,B,E), whereas GABAergic synaptic signaling was not significantly different between the two groups (Figure 4C,D,F). Based on the median GSVA score, we divided each spot into four categories of high and low glutamatergic/GABAergic and counted their proportions in the two groups. The proportion of high glutamatergic spots (68.3%) in the DSS group was more than twice that of the CON group (32.3%), while the proportion of high GABAergic spots (56.6%) in the DSS group was only 13% higher than that in the CON group (43.6%) (Figure 4G,H). Then we further defined the high glutamatergic spot and low GABAergic spot as excitatory spots; vice versa as inhibitory spots. The results showed that the proportion of excitatory spots in the DSS group was nearly twice that of the CON group, and the increased excitatory spots were widely distributed throughout the hippocampus, including CA1, CA3, and DG (Figure 4I). These results suggest that hippocampal excitatory/inhibitory imbalance is a potentially important mechanism of peripheral inflammation‐induced anxiety, and the overactivation of glutamate synaptic signaling mainly mediates the process.

**FIGURE 5:**
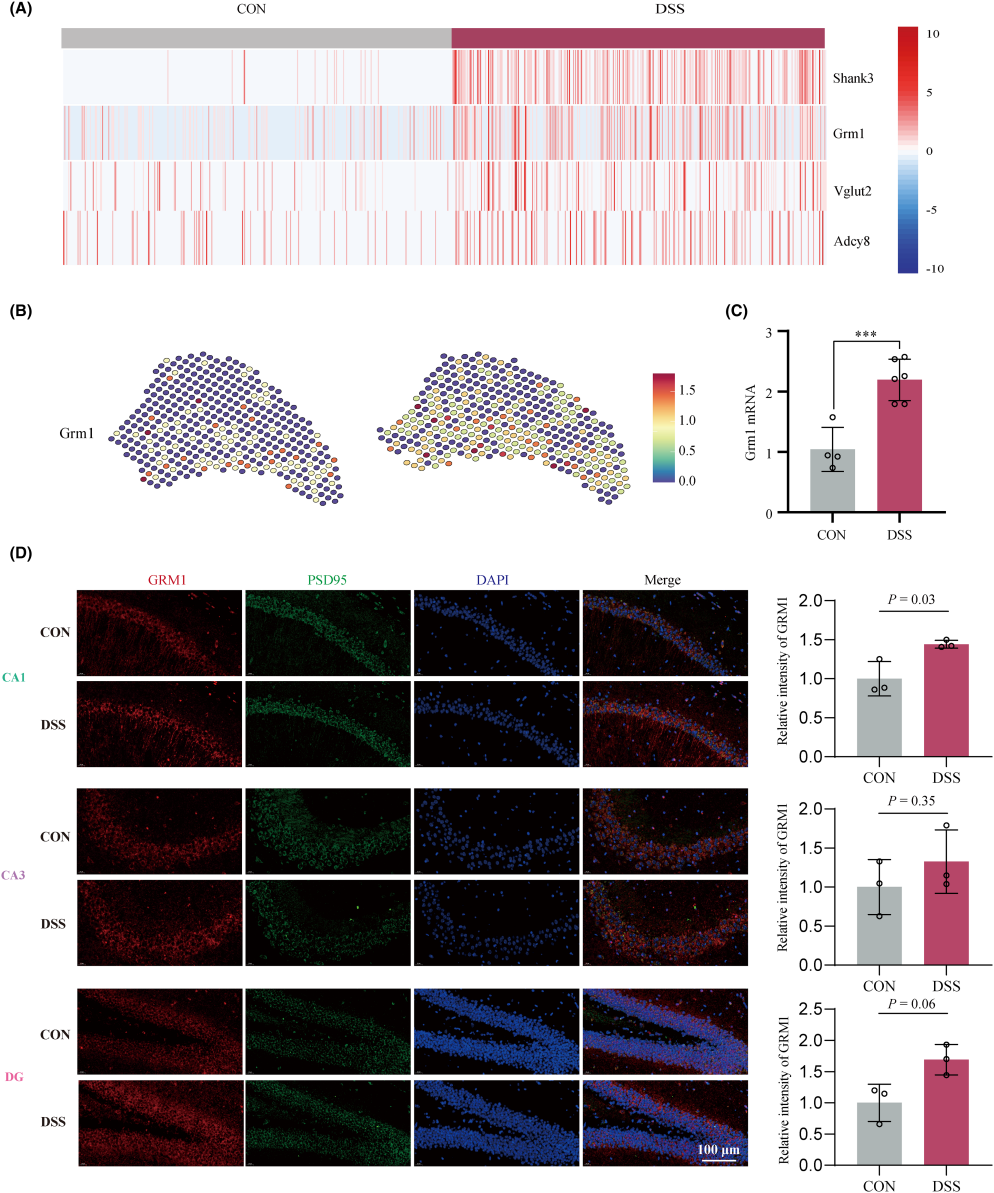
GRM1 was a key molecule in enhanced glutamate synaptic signaling of colitis mice. (A) The heatmap of top differentially genes of glutamatergic synaptic pathway in the DSS group compared with the Control group. (B) GRM1 exhibits a spatially differential expression pattern similar to glutamatergic synaptic signaling. (C) Statistical results indicate that DSS‐treatment increased the mRNA expression level of Grm1in hippocampus. ***p < 0.001. (4 mice in Control group and 6 mice in DSS group). (D) Representative images and statistical results indicate that DSS‐treatment increased protein expression of GRM1 (Red) in hippocampus (*n* = 3). PSD95 (green) and DAPI (blue) are markers for the postsynaptic membrane and nucleus. Scale bar = 100 μm.

Then we examined the expression signatures of core differential genes between the two groups in the glutamate synaptic signaling. Among the top differential genes (Fold Change ≥1.5) (Figure 5A, Figure S8A), *Grm1* and *Shank3* (Src homology 3 and multiple ankyrin repeat domains 3) have spatially differential expression patterns similar to glutamate synaptic signaling (Figure 5B, Figure S8A). Since numerous studies have reported that GRM1 induces anxiety‐like behaviors in various animal models,^25^, ^26^, ^27^, ^28^ we examined the expression of *Grm1* in the hippocampus. RT‐qPCR (Figure 5C) and IF (Figure 5D) results showed that *Grm1* was significantly upregulated in the hippocampus of colitis mice, suggesting that it may be a critical target of peripheral inflammation‐induced anxiety behavior.

### 3.5 Hippocampal AAV‐Grm1‐shRNA injection ameliorated the anxiety‐like behaviors of DSS‐induced colitis mice

**FIGURE 6:**
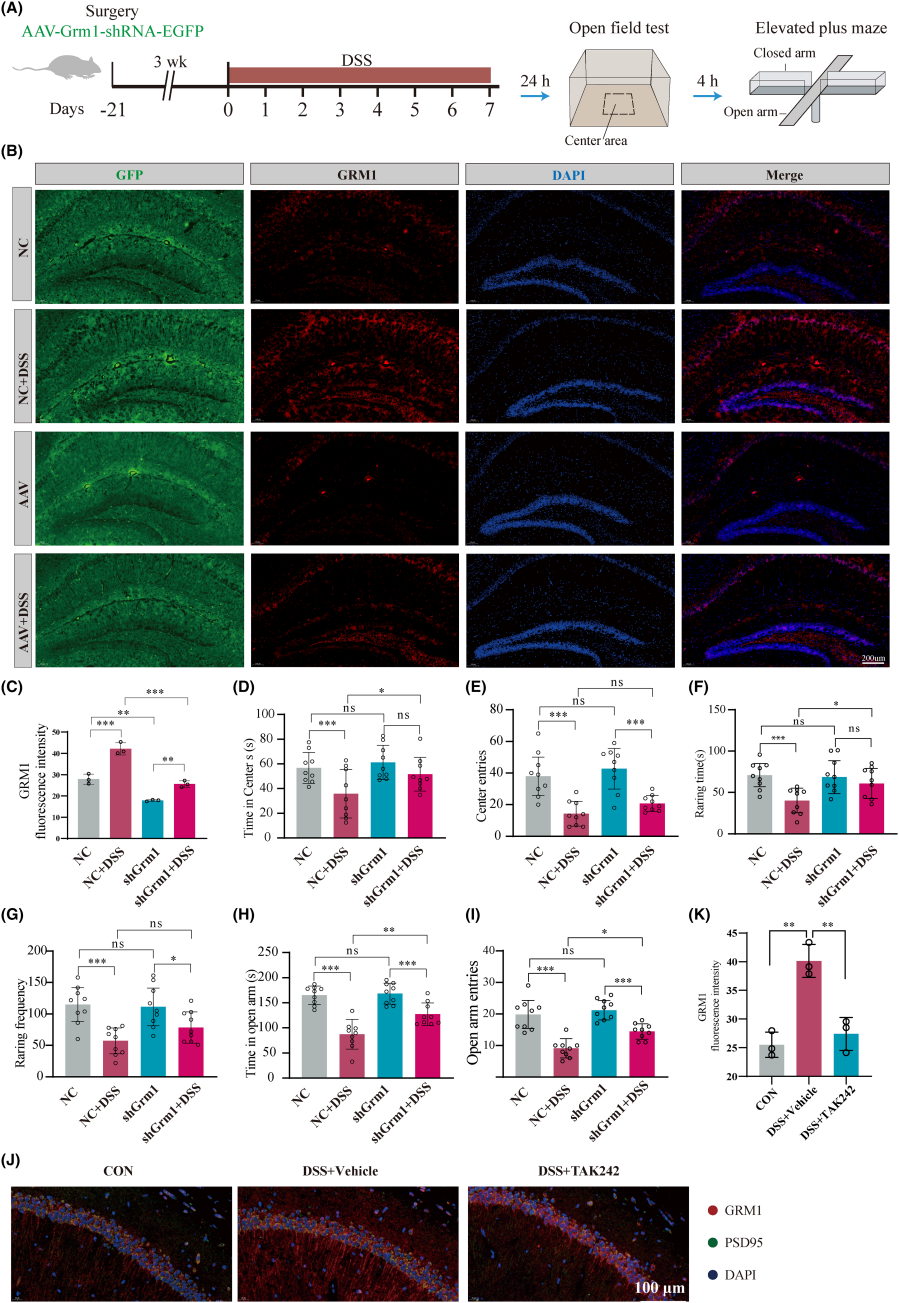
Hippocampal AAV‐Grm1‐shRNA injection ameliorated the anxiety‐like behaviors of DSS‐induced colitis mice. (A) Schematic diagram of the experimental procedure. (B,C) Qualitative evaluation of GRM1 expression following AAV‐scrambled‐shRNA or AAV‐GRM1‐shRNA injection into the hippocampus (*n* = 3). (D) Statistical results indicate AAV‐Grm1‐shRNA increased the time spent in the center area during OFT (*n* = 9). (E) AAV‐Grm1‐shRNA increased center entries during OFT. (F) AAV‐Grm1‐shRNA increased the raring time during OFT. (G) AAV‐Grm1‐shRNA increased the raring frequency OFT. (H) Statistical results indicate that AAV‐Grm1‐shRNA increased time spent in open arms during EPM (*n* = 9). (I) AAV‐Grm1‐shRNA increased entries to open arm during EPM. (J) Representative images showing GRM1 expression in hippocampus. PSD95 (green) and DAPI (blue) are markers for the postsynaptic membrane and nucleus. Scale bar = 100 μm. (K) Statistical results indicate that TAK‐242 increased protein expression GRM1 in hippocampus (*n* = 3). *p < 0.05, **p < 0.01, ***p < 0.001, NS, no significance.

To address this hypothesis, we designed three shRNAs (Figure S9A,B) and cloned them into adeno‐associated virus vector to directly knockdown DSS‐induced *Grm1* expression in the hippocampus to examine the effect of this knockdown on anxiety behavior impact. Initial screening of shRNA candidates in HEK 293 cells showed that shRNA2 had the highest interference efficiency (Figure S9C). Then, stereoscopic injection of AAV‐Grm1‐shRNA into the hippocampus 3 weeks before DSS‐induced colitis model (Figure 6A). After clarifying the inhibitory effect on hippocampal GRM1 (Figure 6B,C; Figure S9D–F), the effect of AAV‐Grm1‐shRNA on anxiety‐like behavior was measured. In comparison with DSS‐induced colitis mice, AAV‐Grm1‐shRNA significantly increased time spent in the center, center entries, raring time, and frequency in OFT (Figure 6D–G). The results of the elevated plus maze experiment also showed that AAV‐Grm1‐shRNA significantly increased time in and more entries to the open arms (Figure 6H,I). More importantly, TAK‐242 significantly reversed the upward trend of GRM1 in the hippocampus of DSS‐induced mice (Figure 6J,K).

## 4 DISCUSSION

This study, based on spatial transcriptome and virus interference, has found that the excitatory‐inhibitory imbalance in the hippocampus in DSS‐induced colitis mice was caused by the overactivation of glutamatergic synapses rather than the inhibition of GABAergic synapses. Multiple enrichment analyses based on differential genes all pointed to glutamatergic synaptic signaling. In contrast, marker gene scores further confirmed the activation of glutamatergic synapses, and GABAergic synapses were not significantly changed between groups. Benefiting from in situ dominance of the spatial transcriptome, we demonstrate that colitis model mice have higher glutamate synapse scores and excitatory spot numbers than controls in three significant subregions of the hippocampus, suggesting that the excitatory‐inhibitory imbalance induced by peripheral inflammation is widespread in the hippocampus.

Interestingly, some colitis animal models also observed increased hippocampal neuronal excitability.^29^ One study used pentylenetetrazole (PTZ: a GABA antagonist) intravenously in TNBS‐induced colitis rats, to induce clonic seizures. The results showed that the susceptibility of colitis rats to PTZ episodes was significantly increased and was closely related to the severity and progression of intestinal inflammation. The hippocampal slices showed an increase in spontaneous burst discharges during the interictal period following 4‐aminopyridine application, suggesting that TNBS‐induced colonic injury significantly increased the intrinsic excitability of rat hippocampal neurons.^29^ Another study using the same animal model showed that four‐day TNBS induction significantly enhanced Schaeffer collateral‐induced excitatory field potentials in the CA1 radial layer of rat hippocampal slices.^30^ These data are in good agreement with our results because the majority of excitatory neurotransmission in the hippocampus relies on glutamatergic synapses.^31^ At the same time, these results also suggest that the increased excitability of hippocampal neurons is a common hub for multiple factors that cause anxiety.

We also found that hippocampal glutamate metabotropic receptor *Grm1* has a spatially differential expression pattern similar to glutamate synaptic signaling, and interfering with *Grm1* expression can significantly improve anxiety behavior in colitis mice.

Glutamate receptors include three groups of metabotropic receptors (mGluR1‐8) and four types of ionotropic receptors (NMDA, AMPA, δ, and kainite),^32^, ^33^ of which ionotropic receptors NMDA and metabotropic receptors mGluRs are widely studied.^34^ The metabotropic glutamate receptor mGluRs family has 8 members, which can be divided into three groups according to their structure and function, namely Group I composed of mGluR1 and mGluR5; Group II composed of mGluR2 and mGluR3; and mGluR4, mGluR6, Group III consisting of mGluR7 and mGluR8. Among them, mGluR1, mGluR2, mGlu3 and mGluR5 have been reported to play a role in anxiety behavior by preclinical studies.^35^ For example, the mGluR1 receptor antagonist JNJ16259685 can inhibit the anxiety behavior of rats in the lick suppression test.^25^ Another selective and competitive mGluR1 antagonist AIDA can significantly improve the anxiety performance of drinking conflict‐induced‐anxiety rats in OFT and EPM.^36^ Our results demonstrate for the first time that inhibition of hippocampal mGluR1 (GRM1) improves anxiety‐like behavior in DSS‐induced colitis mice.

Another intriguing observation is that *Shank3*, encoding the SHANK3 protein, shows the most significant trend of changes, along with a spatial distribution consistent with the overall alterations in glutamatergic signaling (similar to *Grm1*). SHANK3 belongs to a family of SHANK proteins (SHANK1‐3), all of which directly bind SAPAP to form the PSD95/SAPAP/Shank complex.^37^ This core of proteins is thought to function as a scaffold, orchestrating the assembly of the macromolecular postsynaptic signaling complex at glutamatergic synapses.^38^ More importantly, *Shank3* has been identified as one of the few genes whose mutations can lead to autism. Mice with *Shank3* gene deletions exhibit self‐injurious repetitive grooming and deficits in social interaction.^39^ However, there are few reports on the relationship between SHANK3 and anxiety. Due to its ability to form physical contact with GRM1 through the synaptic scaffold formed by PSD95‐SAPAP‐SHANK3,^40^ further research is warranted to investigate its role in central glutamate signaling transduction induced by peripheral inflammation.

Additionally, spatial transcriptome analysis also found that other receptors such as NMDAR2C/D, NMDAR3A, and AMPA receptor GRIK4/5 were highly increased in the hippocampus of DSS‐induced colitis mice. Considering the reports and potential of some NMDA receptor antagonists in improving patients with refractory generalized anxiety and/or other anxiety disorders,^34^, ^41^, ^42^ this study cannot rule out the possibility that these glutamate receptors are involved in anxiety‐like behaviors in mice with colitis.

Although the pathogenesis of colitis is largely unknown, the inflammatory cascade and the release of multiple pro‐ and anti‐inflammatory cytokines and chemokines that accompany colonic tissue damage are its most characteristic features.^43^ To clarify the contribution of colonic inflammation to changes in hippocampal synaptic plasticity, we used TAK‐242 to inhibit colon inflammation to observe whether it could improve anxiety behavior in DSS‐induced mice and regulate the expression of glutamate synapse‐related proteins. TAK‐242 (resatorvid) is a novel cyclohexene derivative that selectively binds to TIR domain of TLR4, disrupting its ability to interact with its adaptor molecules and inhibiting TLR4‐mediated multiple cytokine production.^44^ TAK‐242 can suppress the LPS‐induced production of TNF‐α, IL‐1β, IL‐6, and NO at IC50 values ranging from 11 to 33 nM.^45^ In addition, differences in species do not greatly affect the inhibitory effects of TAK‐242 on cytokine production.^45^ Recent findings have indicated that TAK‐242 dose‐dependently alleviated DSS‐induced colitis symptoms and colonic lesions by downregulating TLR4 and JAK2/STAT3 mRNA and protein expression and increasing JAK2/STAT3 phosphorylation.^20^ Consistent with the above, 10 mg/kg TAK‐242 (maximum reported effective dose) markedly reduced the levels of IL‐1β, IL‐6, and TNF‐α in colon and serum of DSS‐induced colitis mice. More importantly, we demonstrate for the first time that TAK‐242 ameliorates anxiety‐like behavior in DSS‐induced colitis mice and suppresses hyperactivated glutamate synaptic signaling. These results provide further evidence for a brain–gut mechanism by which peripheral inflammation triggers emotional abnormalities.

There are some limitations within our study. First, our study only observed and validated the expression level of glutamate synaptic receptors, lacking electrophysiological data on the intrinsic excitability of hippocampal neurons and the level of synaptic transmission in projection neurons. Second, some low abundance genes may not be well represented in Spatial transcriptome, which may result in biases inherent in incomplete annotations. Thus, other techniques (e.g., single cell sequencing) may be necessary. Last but not at the least, to better understand the relationship of anxiety‐behavior and the expression of GRM1, the concentration of TAK‐242 in serum and cerebral should be monitor in the future research.

In conclusion, our study established DSS‐induced colitis mice's spatial gene expression profile. It confirmed an excitatory‐inhibitory imbalance in the hippocampus caused by hyperactivation of glutamate synapses in colitis mice. We also demonstrate for the first time that the hippocampal glutamate metabolizing receptor GRM1 is an important central target of colitis‐induced anxiety behaviors. Inflammation inhibitor TAK‐242 can significantly inhibit the expression of hippocampal GRM1 and significantly improve anxiety‐like behavior in colitis mice. These results will better understand the bidirectional brain–gut relationship in inflammatory bowel disease‐associated psychiatric disorders.

## AUTHOR CONTRIBUTIONS

Qiao‐Feng Wu had the original idea, designed the study and edited the manuscript. Jun‐Meng Wang wrote the manuscript, conducted the experiments, and analyzed the data. Yue‐Mei Wang initially performed the experiments and analyzed the data. Yuan‐Bing Zhu performed experiments and contributed to write the manuscript. Chan Cui, Tong Feng, Qin Huang supplemented experiments, ensured data integrity, and actively contributed to manuscript editing. Shu‐Qing Liu provided valuable support in bioinformatics analysis methods. Shu‐Guang Yu involved in the study discussion.

## FUNDING INFORMATION

This research was supported by the National Natural Science Foundation of China (No. 82174512, 82305412), the National Key R&D Program of China (No. 2022YFC3500703), Innovation Team and Talents Cultivation Program of National Administration of Traditional Chinese Medicine (No. ZYYCXTD‐D‐202003), Fund of Science and Technology Department of Sichuan Province (No. 2022ZDZX0033, 2023NSFSC1817), and the Postdoctoral Foundation of China (No. 2022MD723717).

## CONFLICT OF INTEREST STATEMENT

The authors declare that they have no competing interests.

## CONSENT FOR PUBLICATION

Not applicable.

## Supporting information


Figure S1.

Figure S2.

Figure S3.

Figure S4.

Figure S5.

Figure S6.

Figure S7.

Figure S8.

Figure S9.

Table S1.

## Data Availability

The datasets used and/or analyzed during the current study are available from the corresponding author on reasonable request.
