# Supplementary figures and images for: Peripheral inflammation triggering central anxiety through the hippocampal glutamate metabolized receptor 1
Source: CNS Neurosci Ther. 2024 Apr 26;30(4):e14723. doi: 10.1111/cns.14723 (PMC11053250; doi:10.1111/cns.14723)

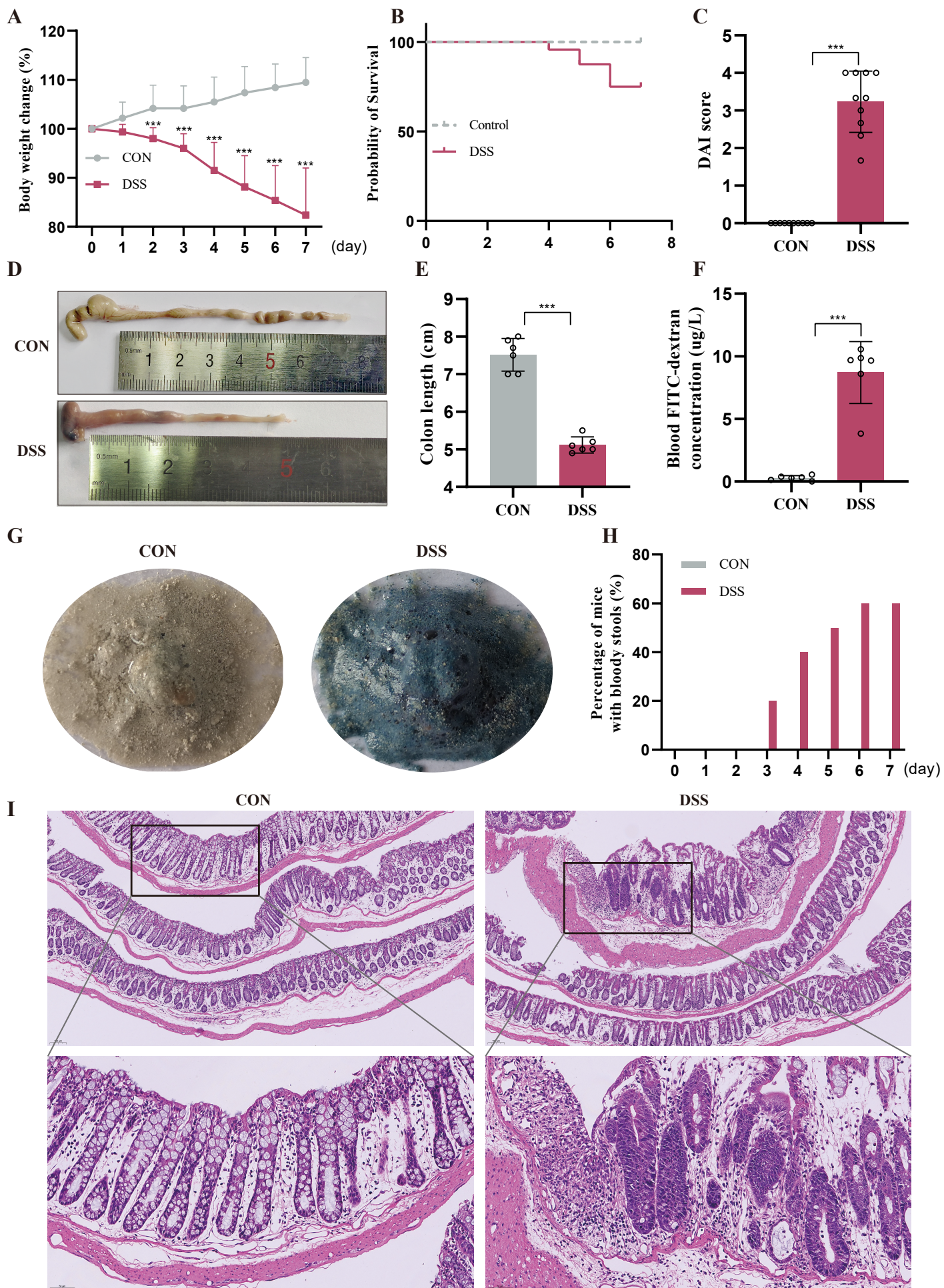

Supplement: Supplementary file 1 — Figure S1. [file CNS-30-e14723-s007.pdf]

**A**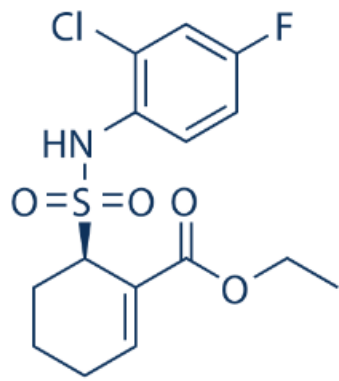**B**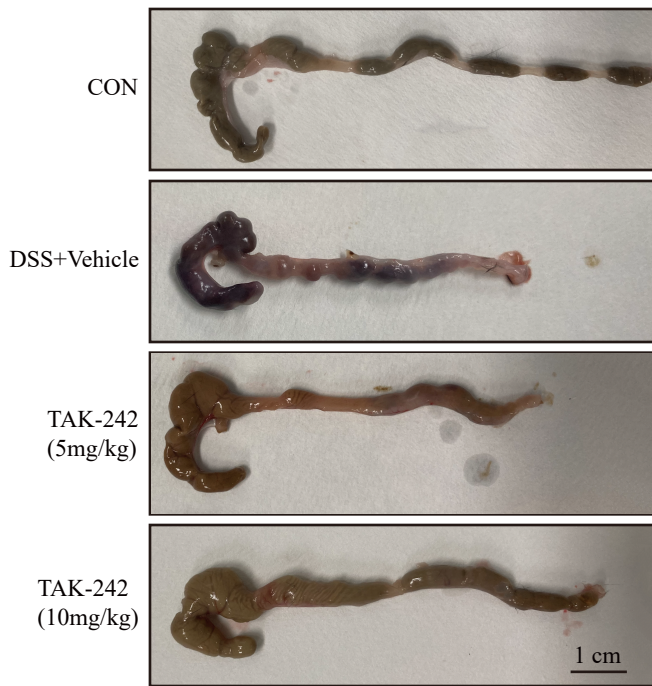**C**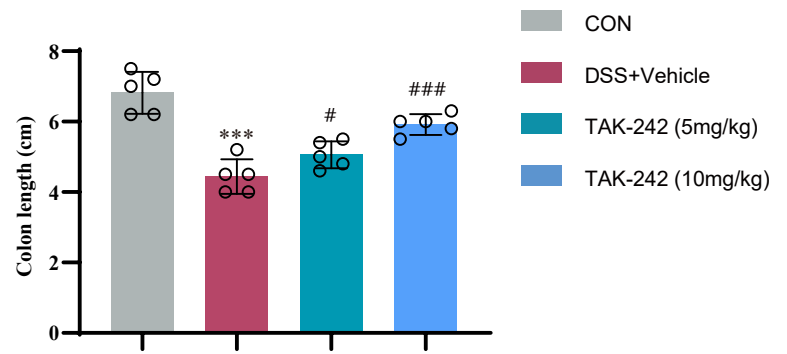**D**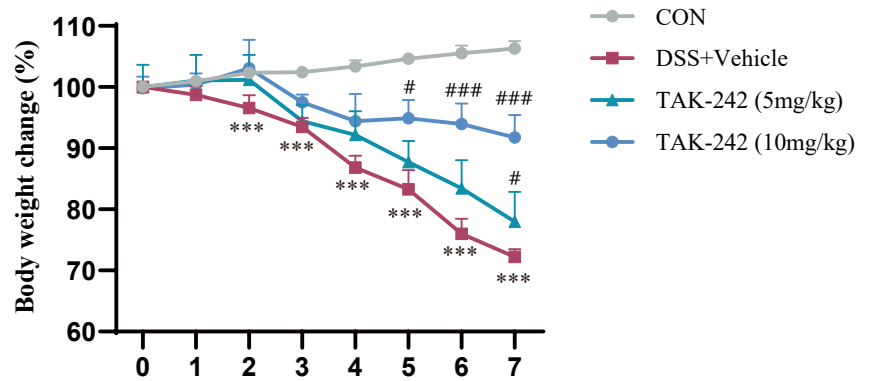**E**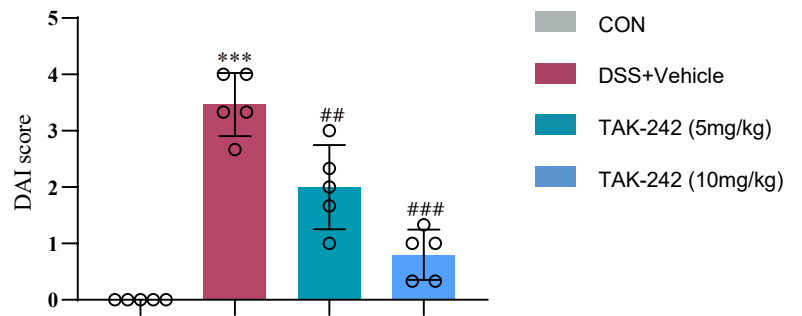**F**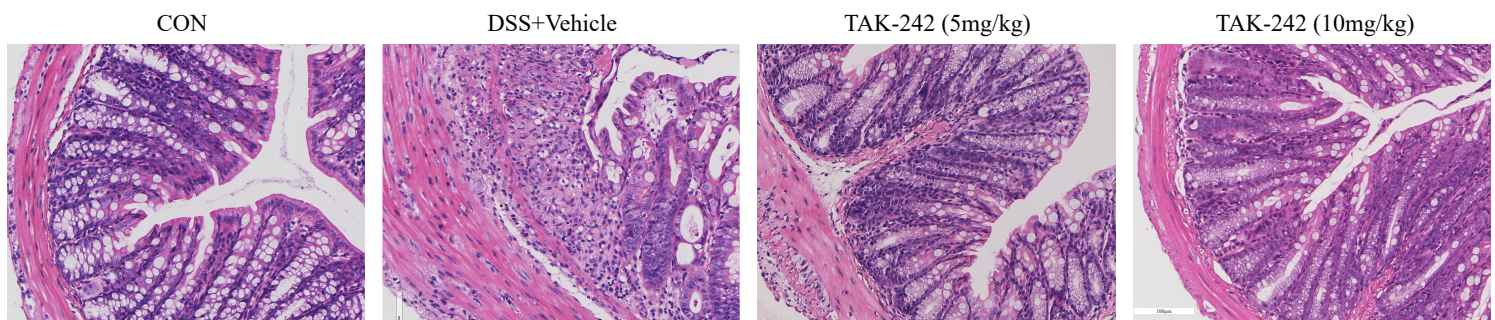

Supplement: Supplementary file 2 — Figure S2. [file CNS-30-e14723-s008.pdf]

**A**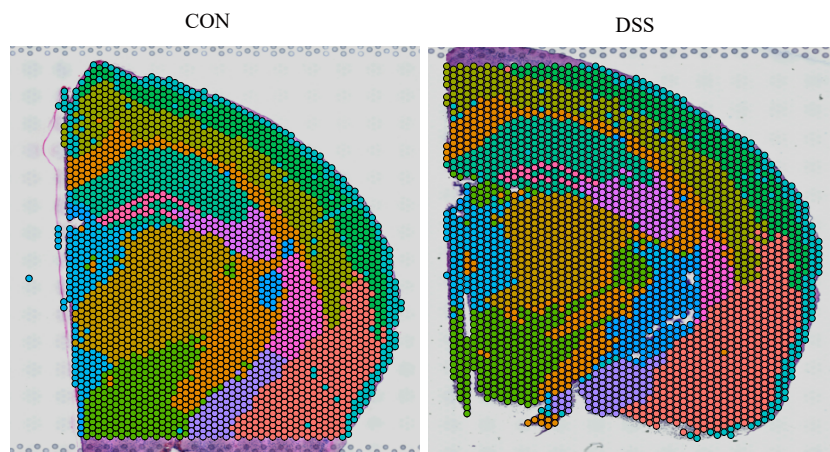**B**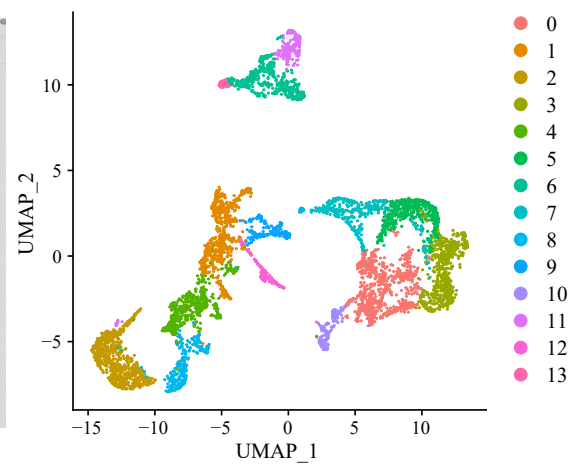**C**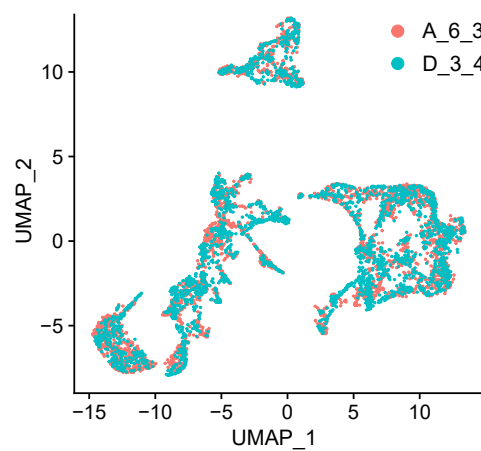**D**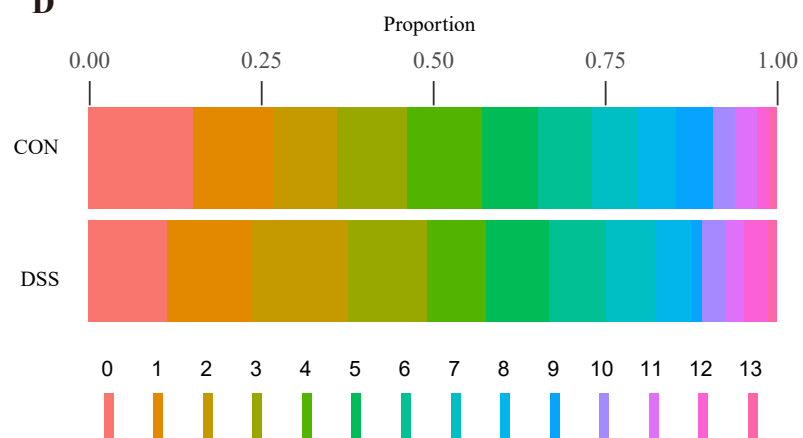**E**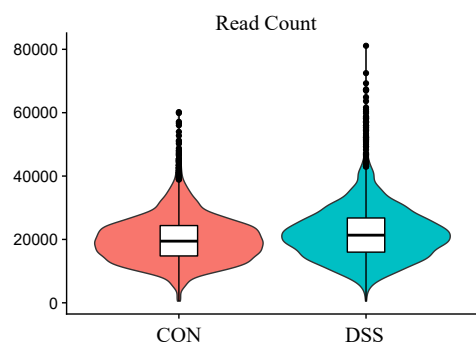**F**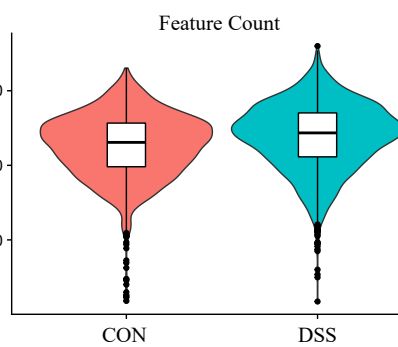**G**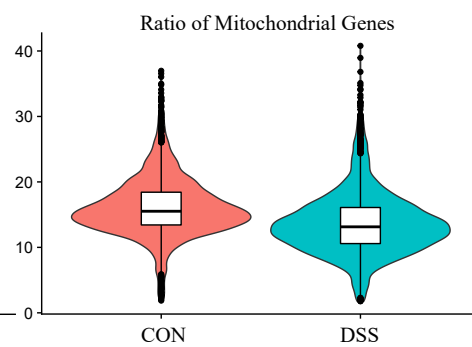

Supplement: Supplementary file 3 — Figure S3. [file CNS-30-e14723-s001.pdf]

0

1

2

3

4

5

6

7

8

9

10

11

12

13

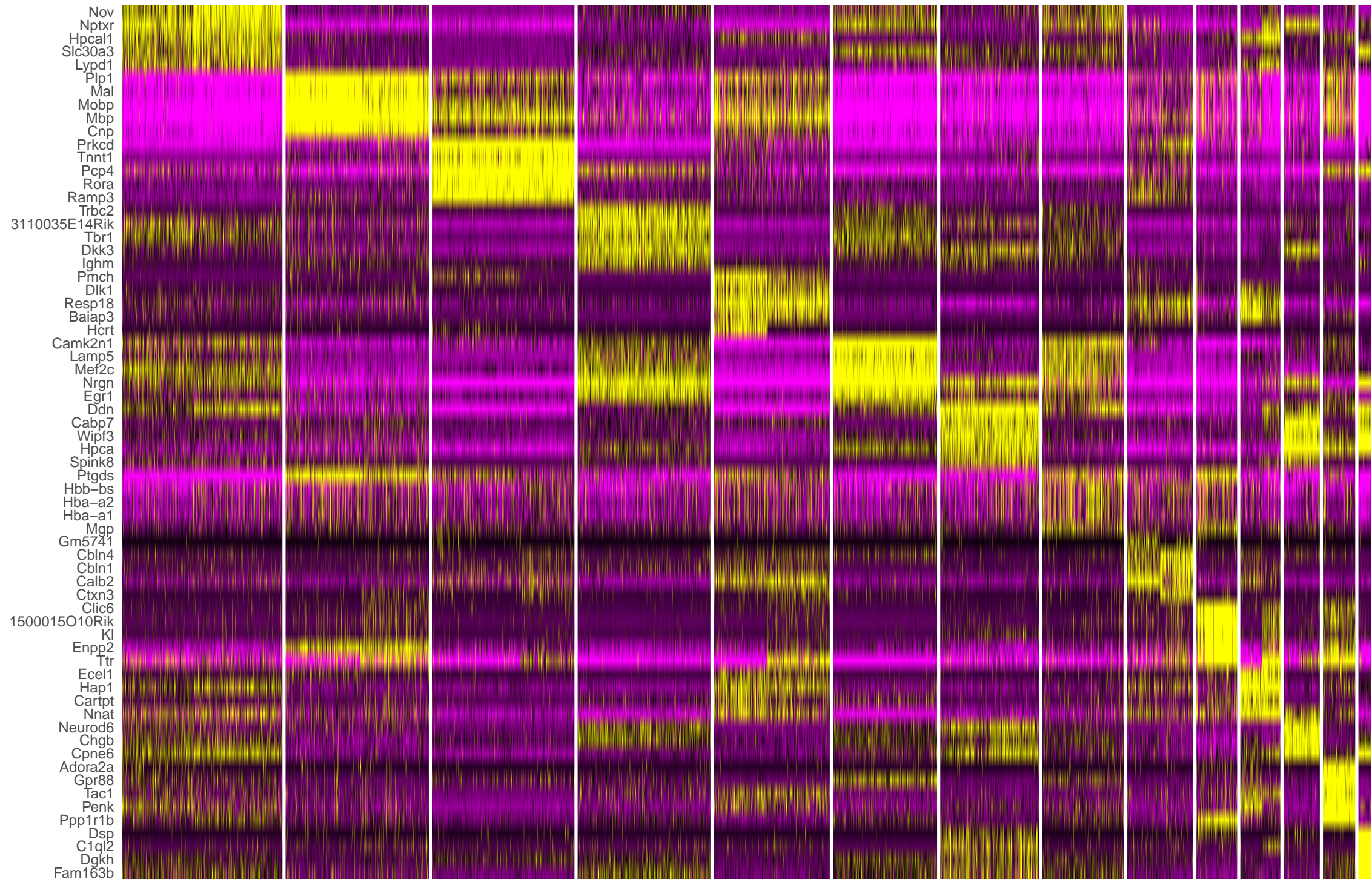

Supplement: Supplementary file 4 — Figure S4. [file CNS-30-e14723-s010.pdf]

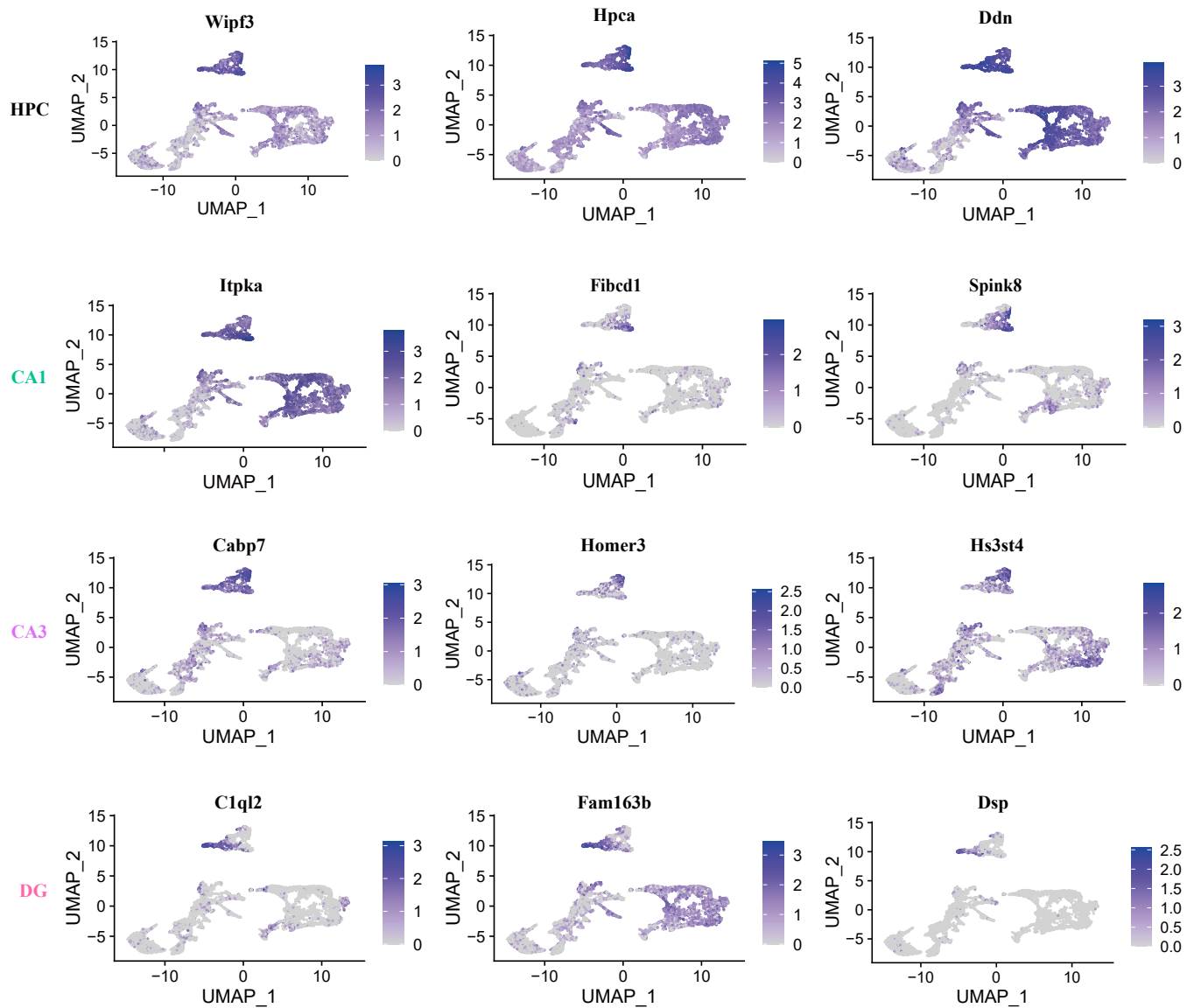

Supplement: Supplementary file 5 — Figure S5. [file CNS-30-e14723-s002.pdf]

HPC

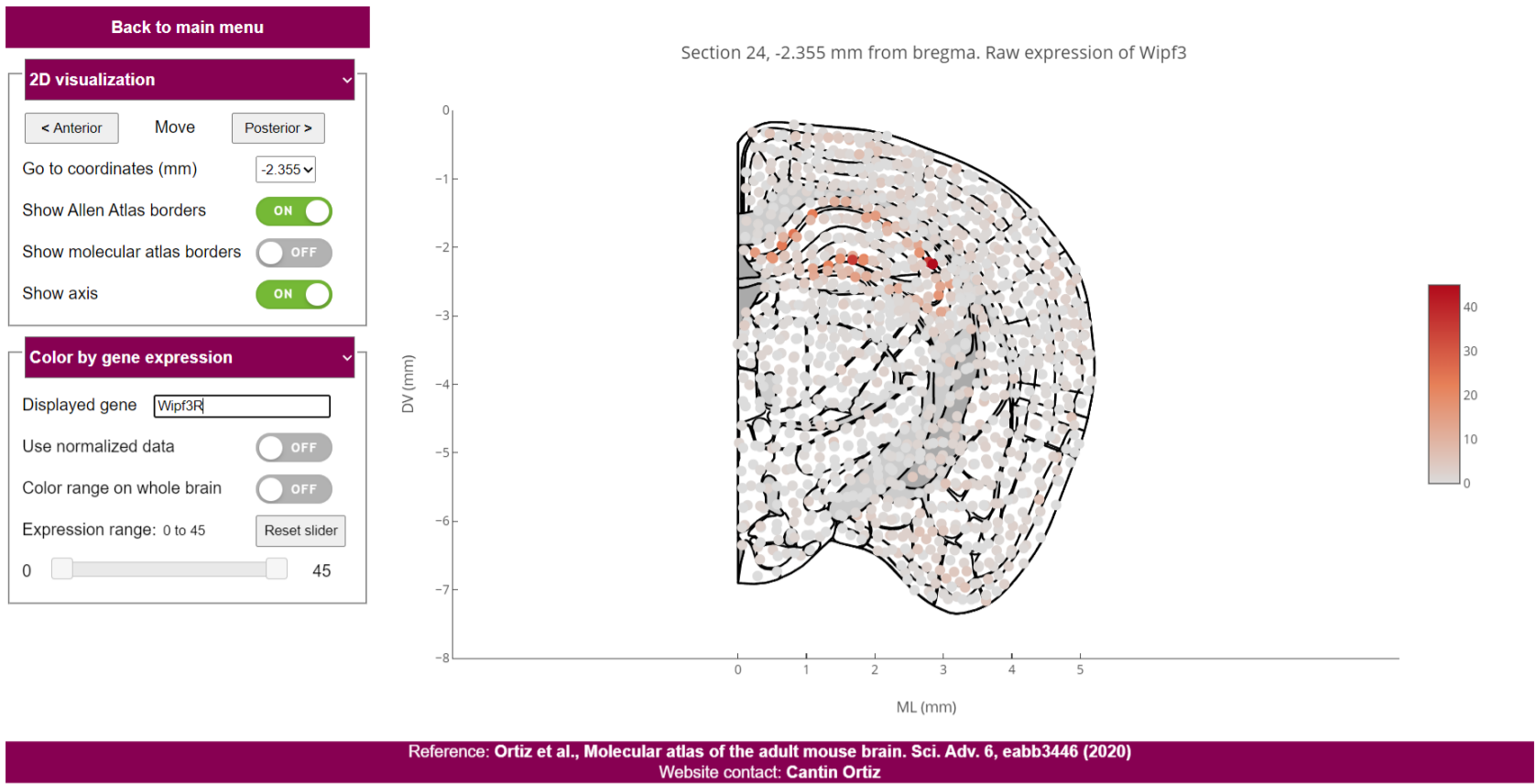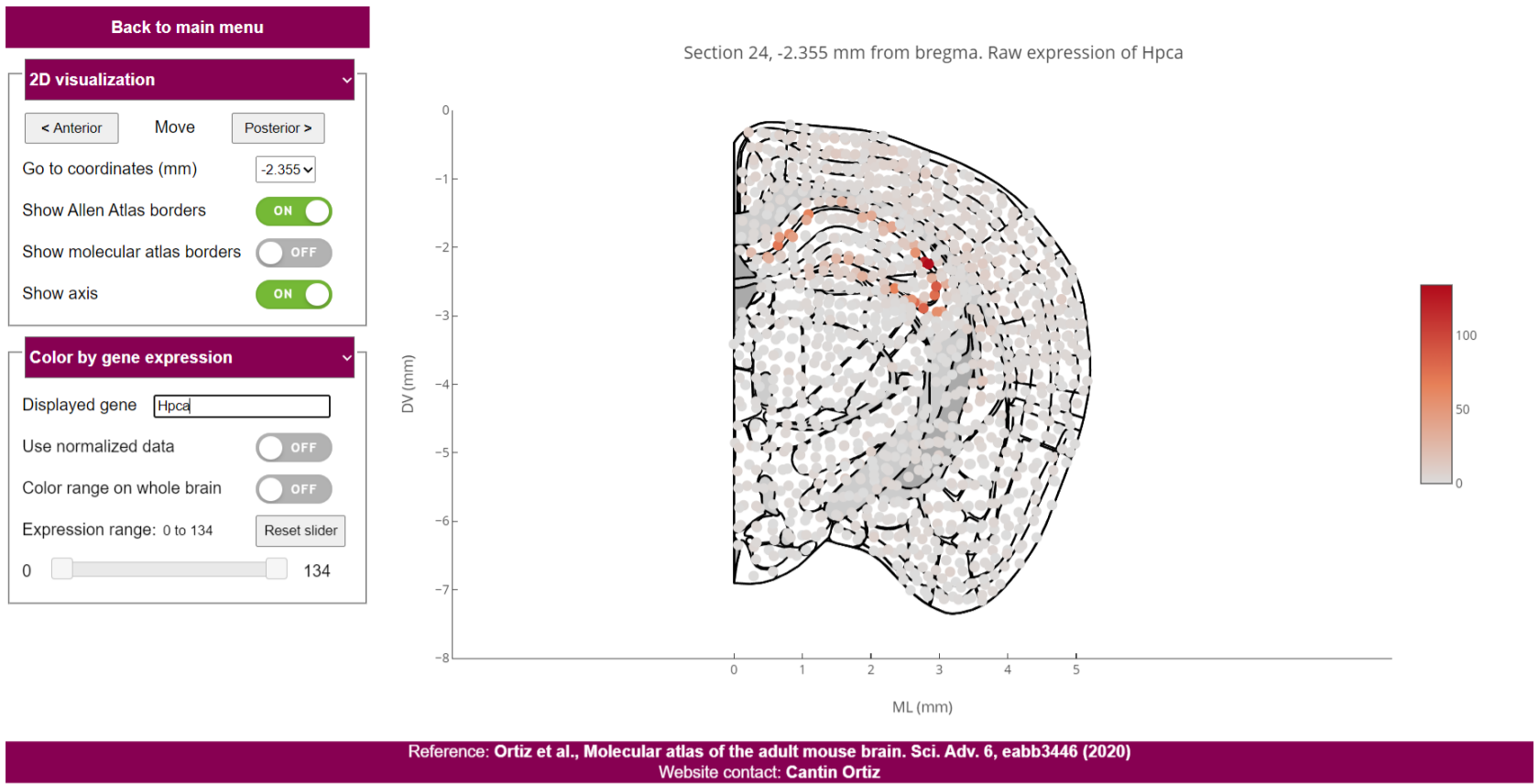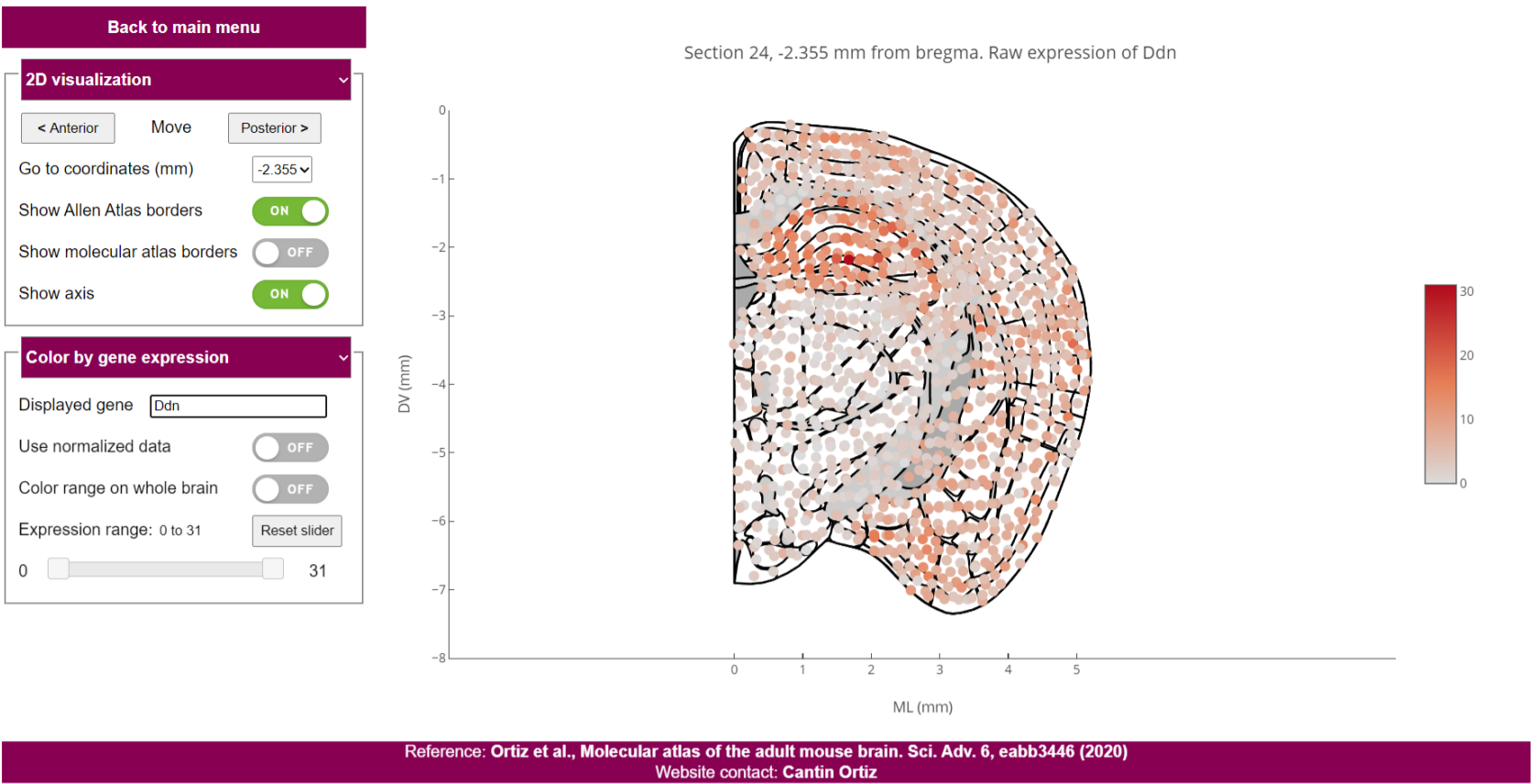

CA1

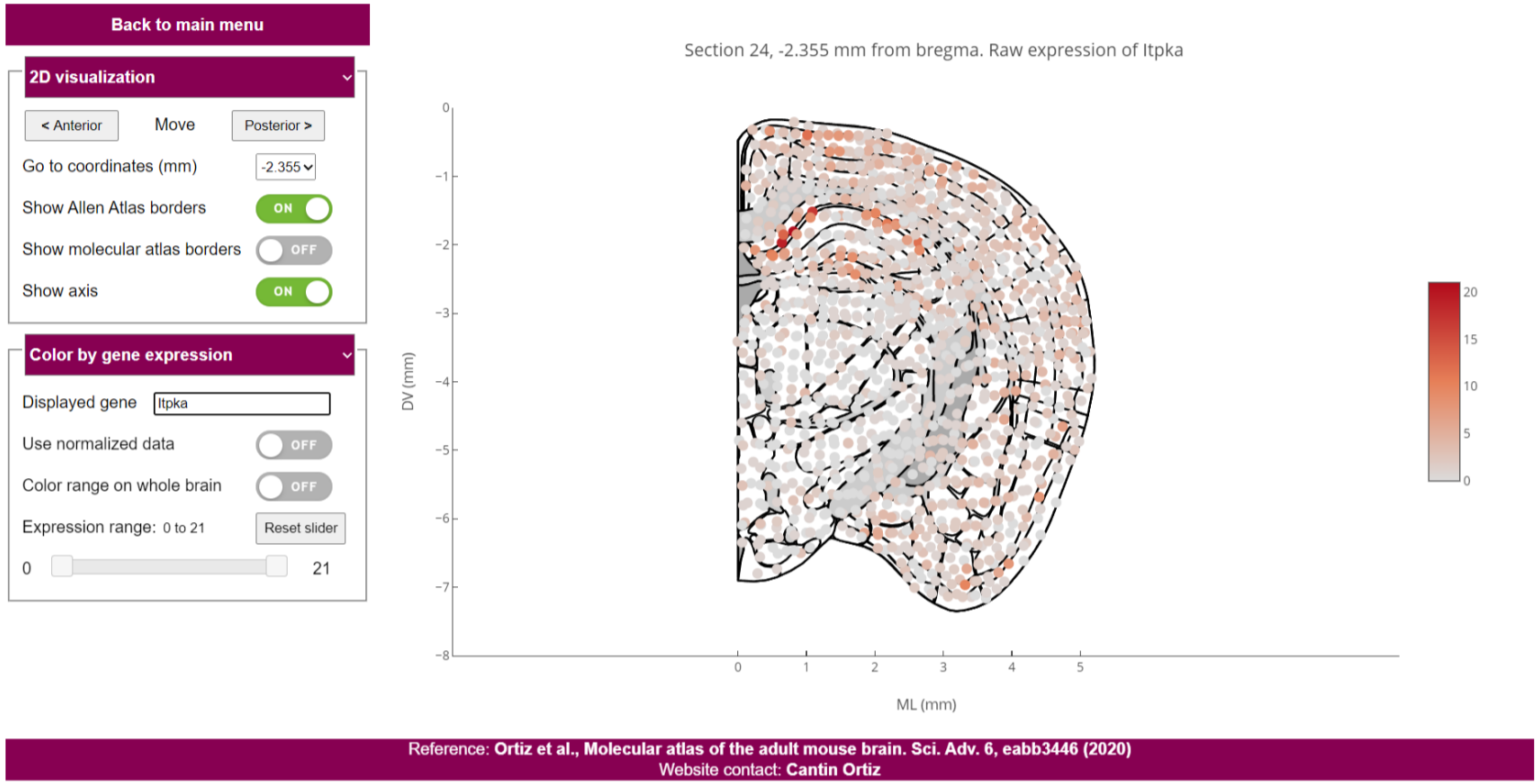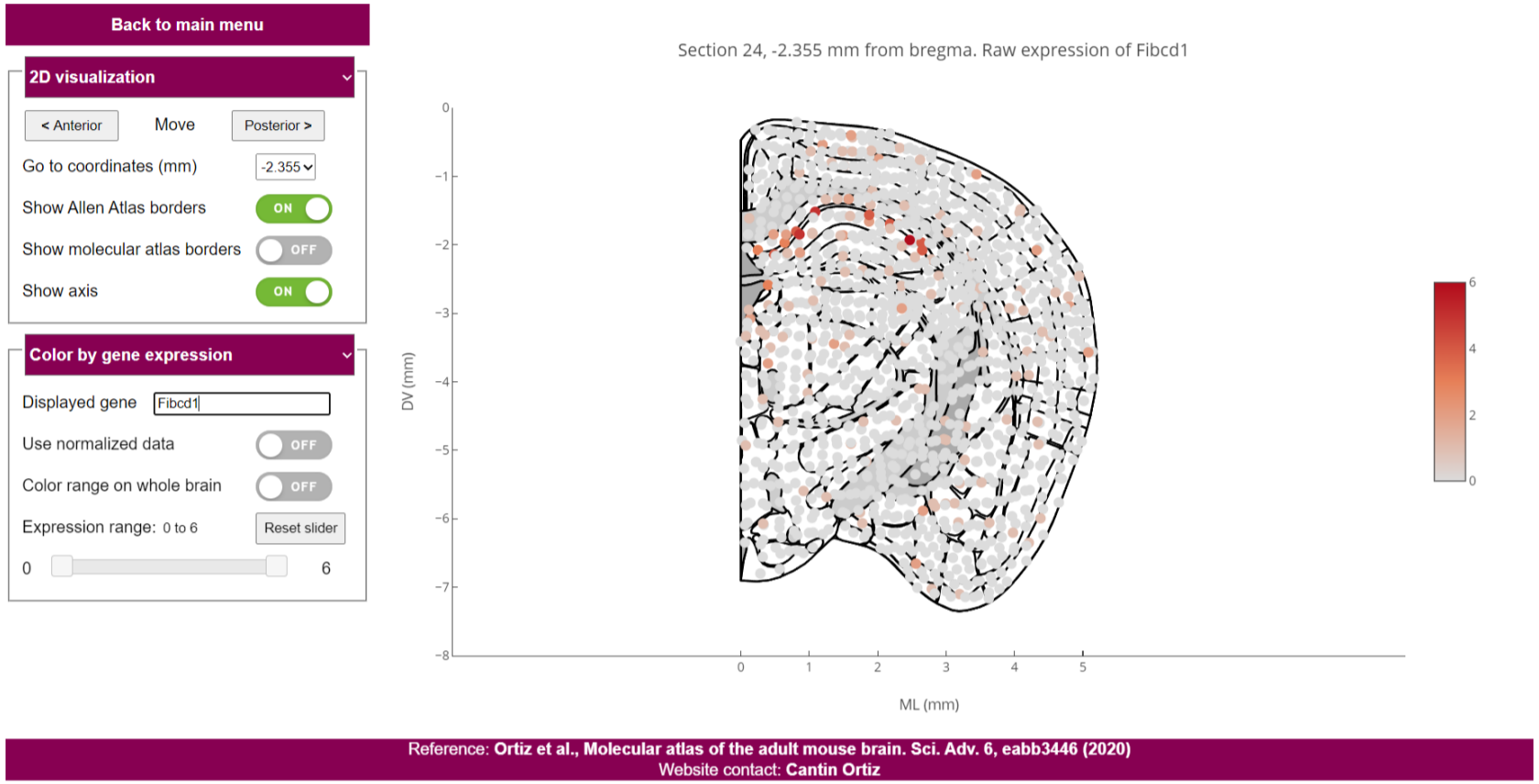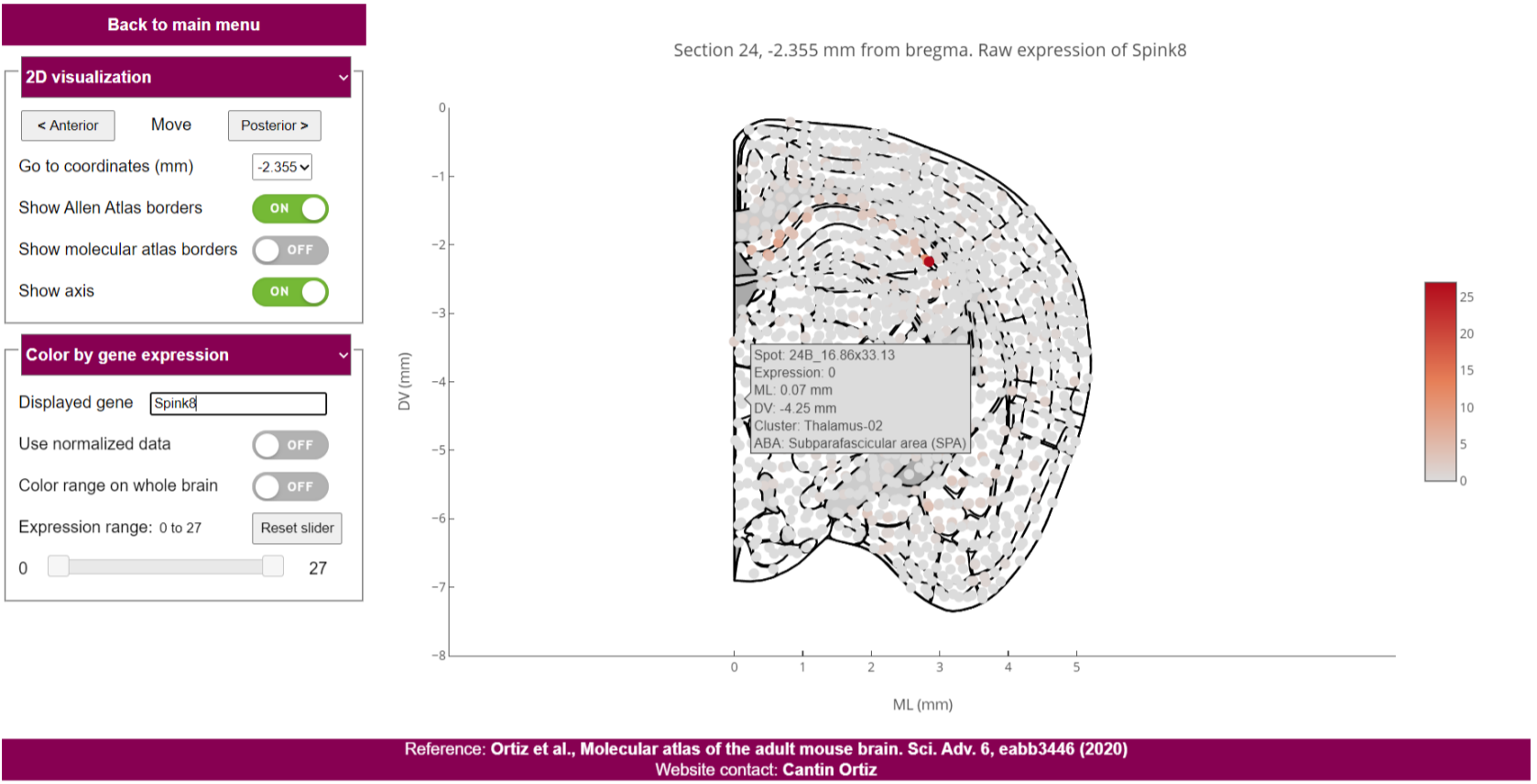

CA3

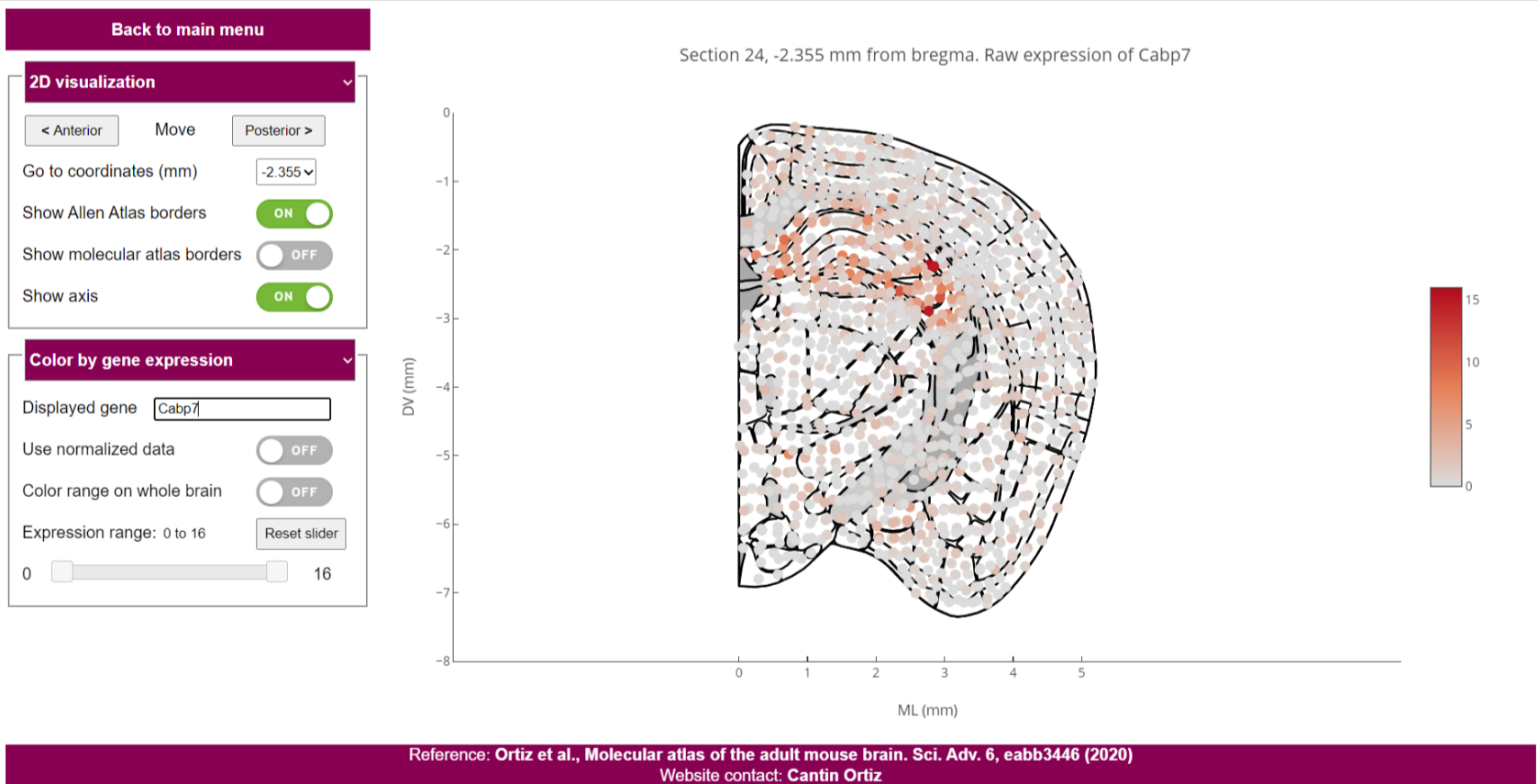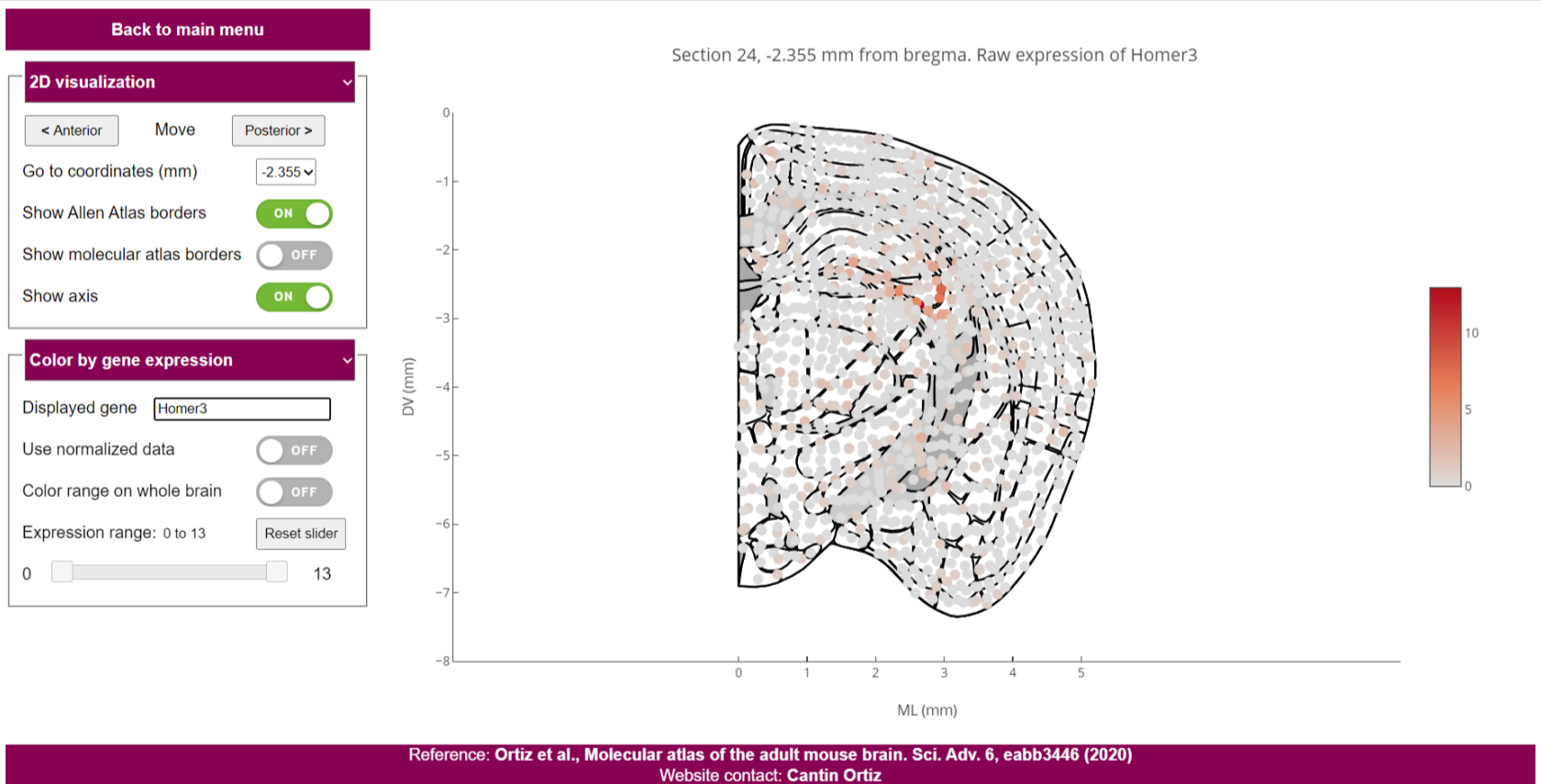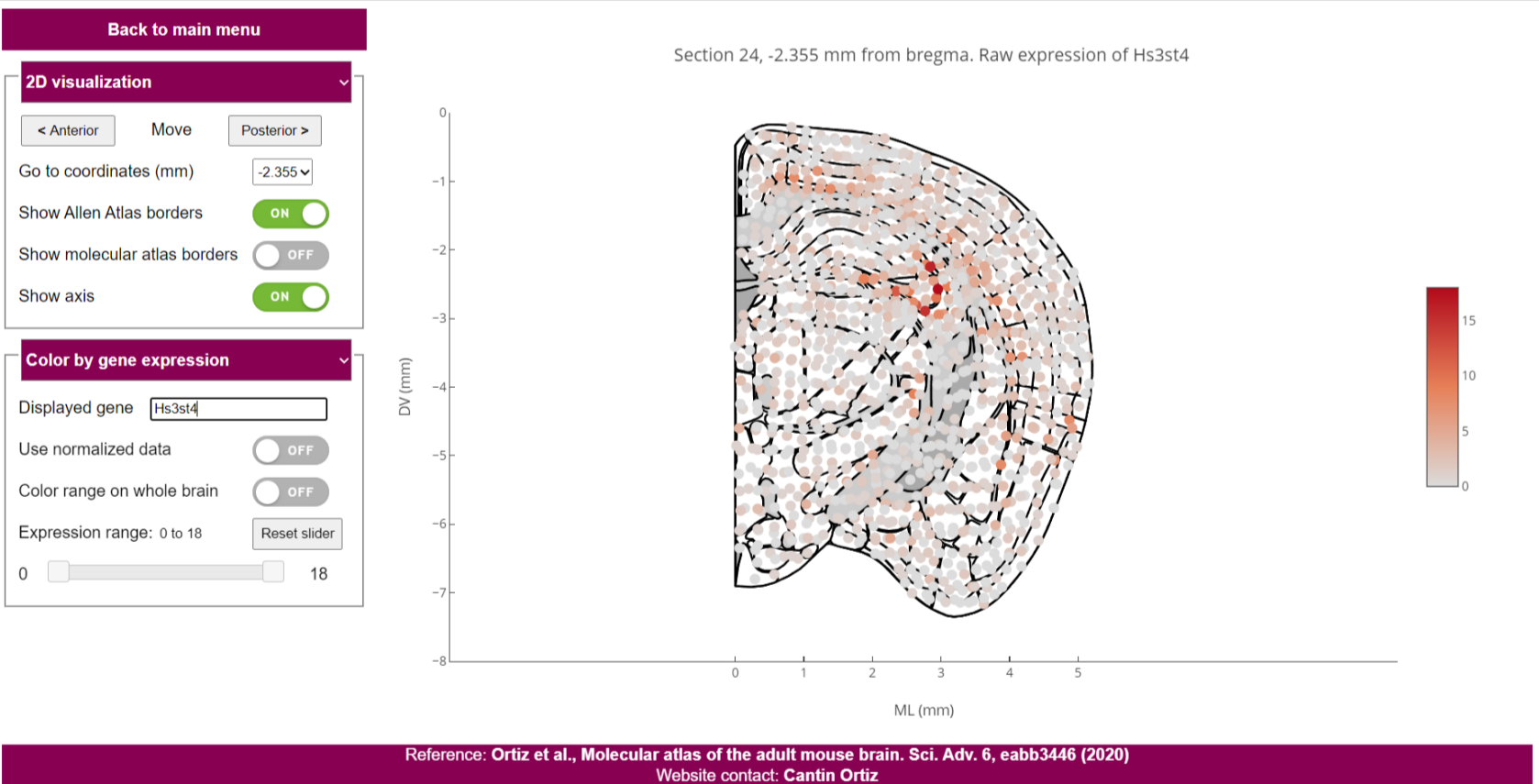

DG

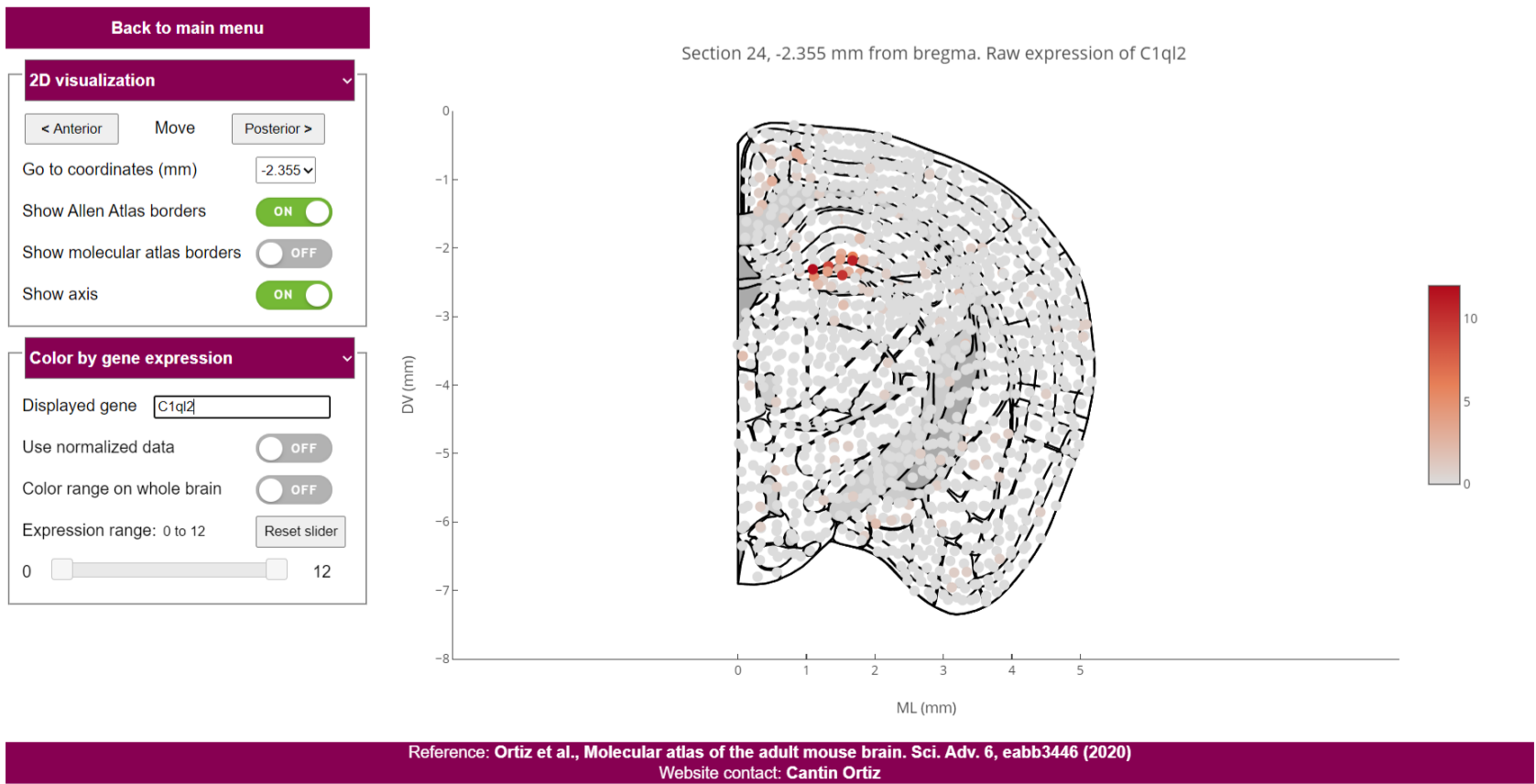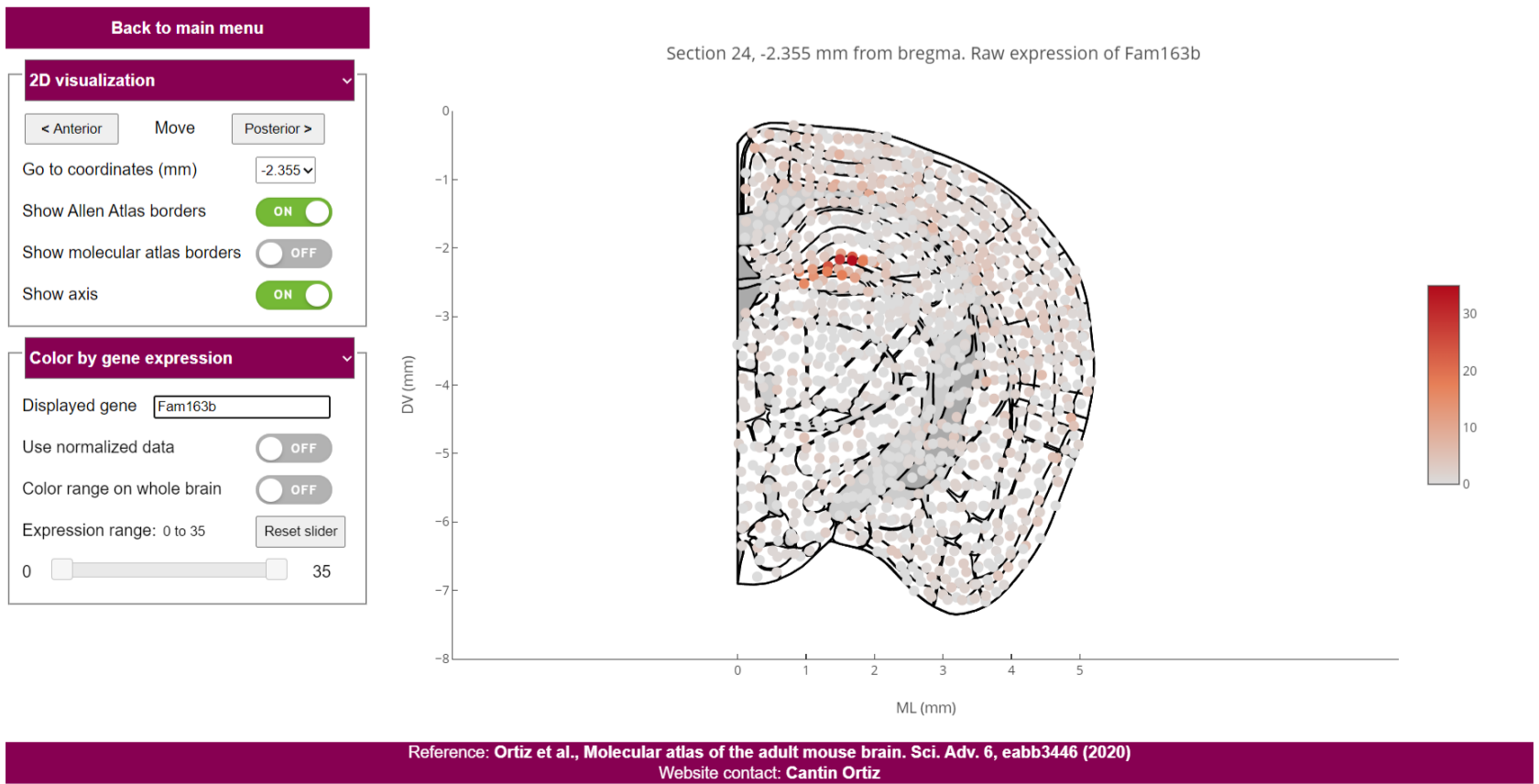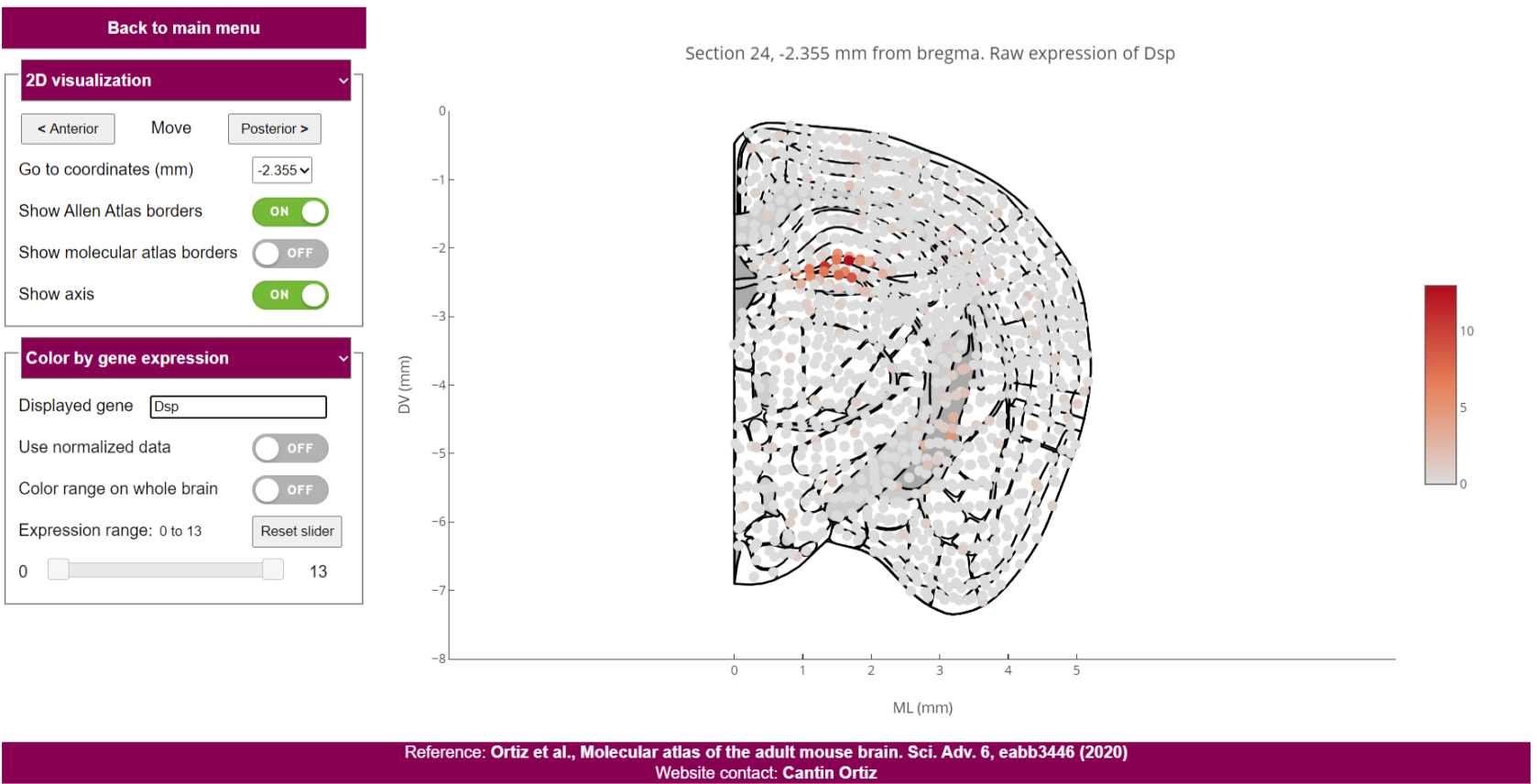

Supplement: Supplementary file 6 — Figure S6. [file CNS-30-e14723-s003.pdf]

**A**

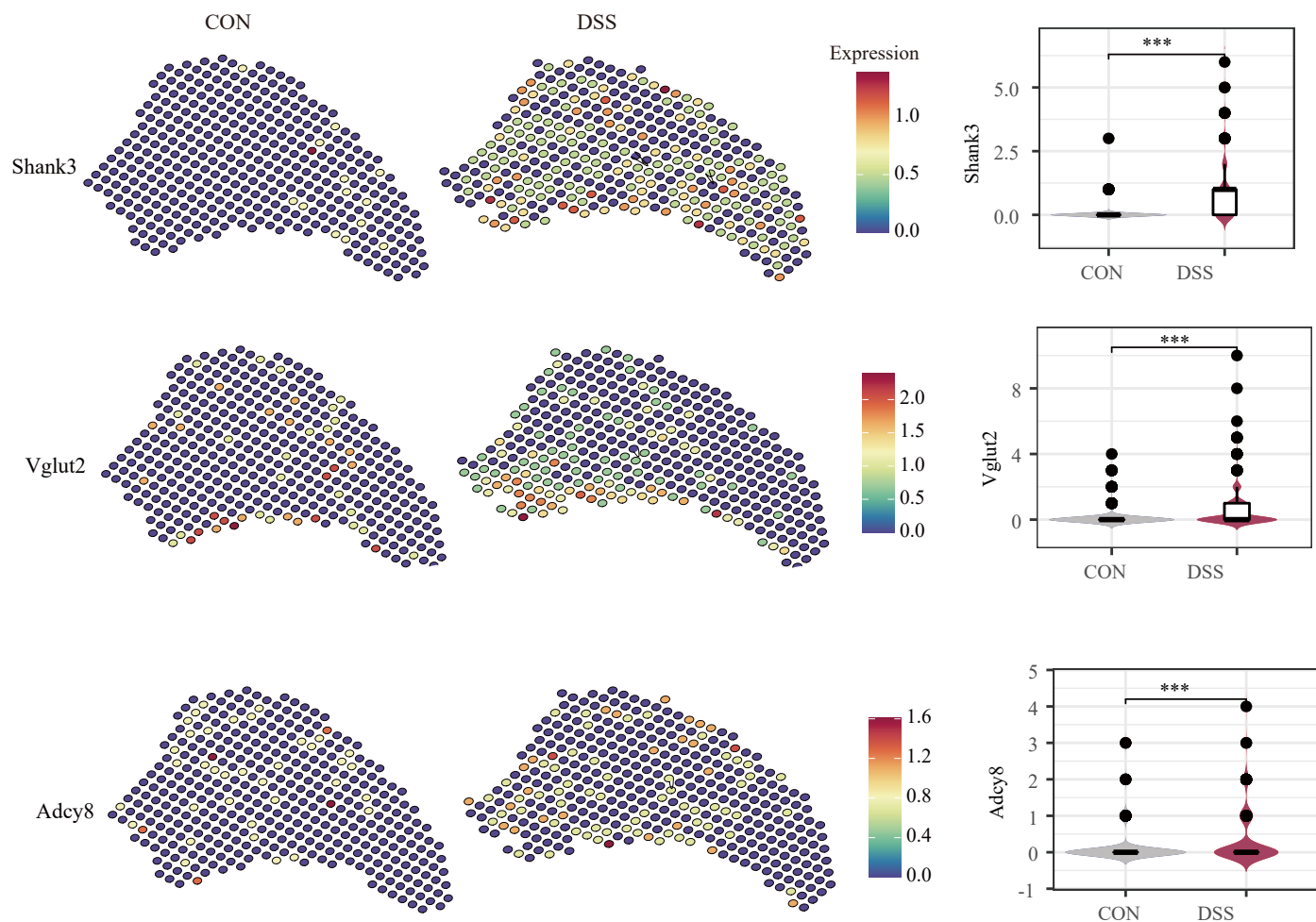

**B**

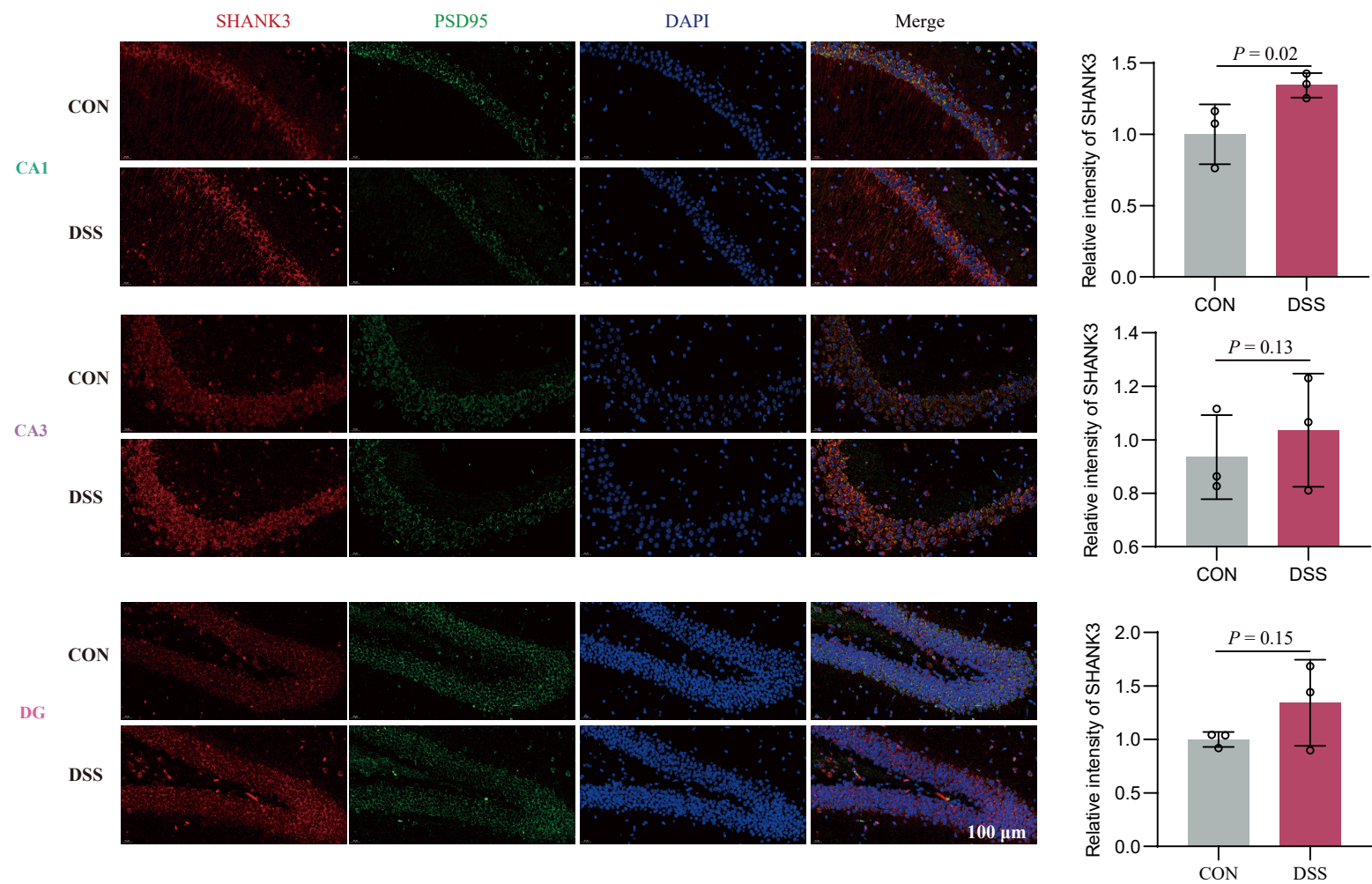

Supplement: Supplementary file 8 — Figure S8. [file CNS-30-e14723-s004.pdf]
