# Supplementary material for: Peripheral inflammation triggering central anxiety through the hippocampal glutamate metabolized receptor 1
Source: CNS Neurosci Ther. 2024 Apr 26;30(4):e14723. doi: 10.1111/cns.14723 (PMC11053250; doi:10.1111/cns.14723)
Supplement: Supplementary file 7 — Figure S7. [file CNS-30-e14723-s006.pdf]

A

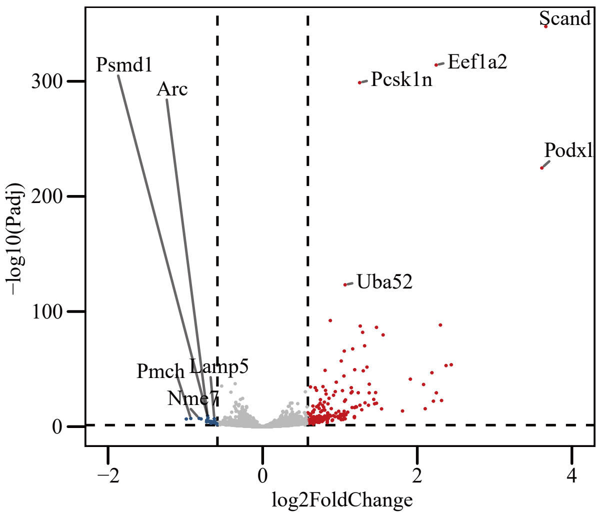

B

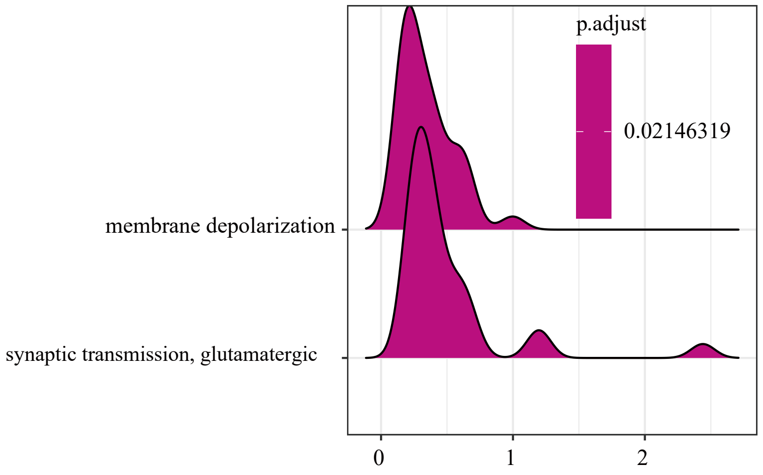

C

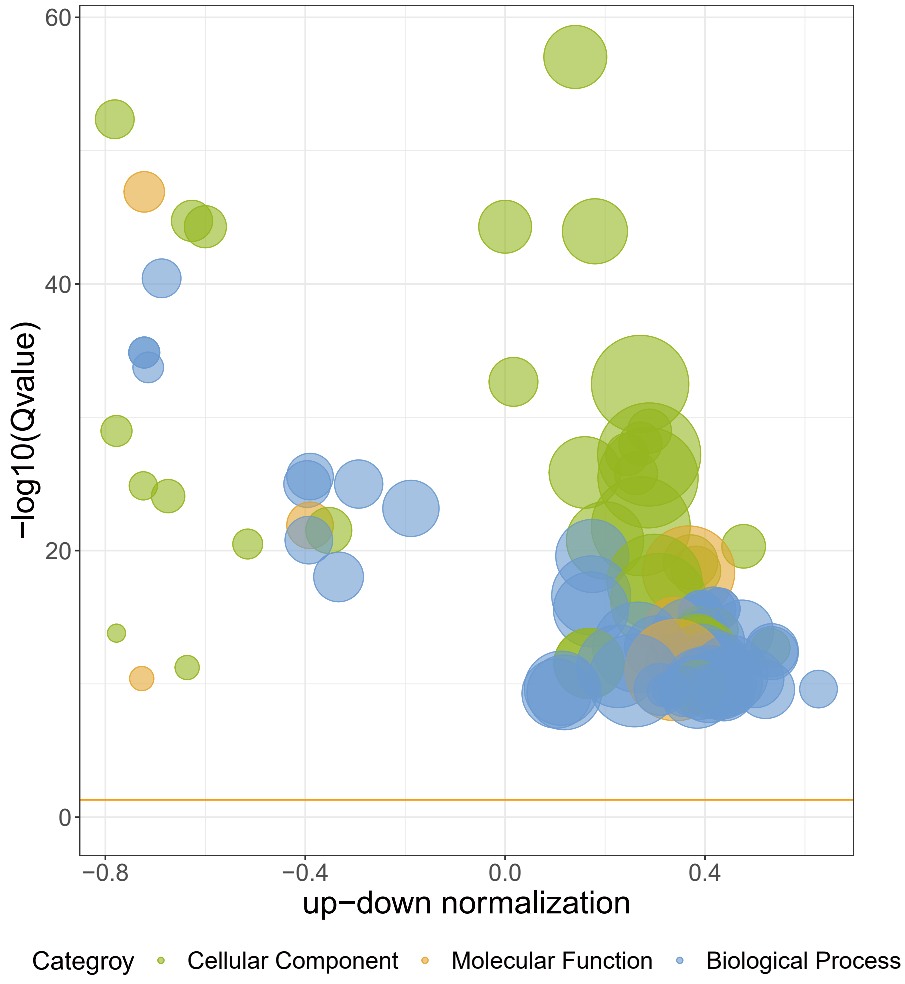

GO term Top 20

ID	Descrption
GO:0045202	synapse
GO:0022626	cytosolic ribosome
GO:0003735	structural constituent of ribosome
GO:0044391	ribosomal subunit
GO:0098794	postsynapse
GO:0005840	ribosome
GO:0030054	cell junction
GO:0002181	cytoplasmic translation
GO:0140241	translation at synapse
GO:0140242	translation at postsynapse
GO:0140236	translation at presynapse
GO:0098793	presynapse
GO:0005737	cytoplasm
GO:0022625	cytosolic large ribosomal subunit
GO:0098984	neuron to neuron synapse
GO:0032279	asymmetric synapse
GO:0005622	intracellular anatomical structure
GO:0014069	postsynaptic density
GO:0005829	cytosol
GO:0099572	postsynaptic specialization
