# Supplementary material for: Peripheral inflammation triggering central anxiety through the hippocampal glutamate metabolized receptor 1
Source: CNS Neurosci Ther. 2024 Apr 26;30(4):e14723. doi: 10.1111/cns.14723 (PMC11053250; doi:10.1111/cns.14723)
Supplement: Supplementary file 9 — Figure S9. [file CNS-30-e14723-s009.pdf]

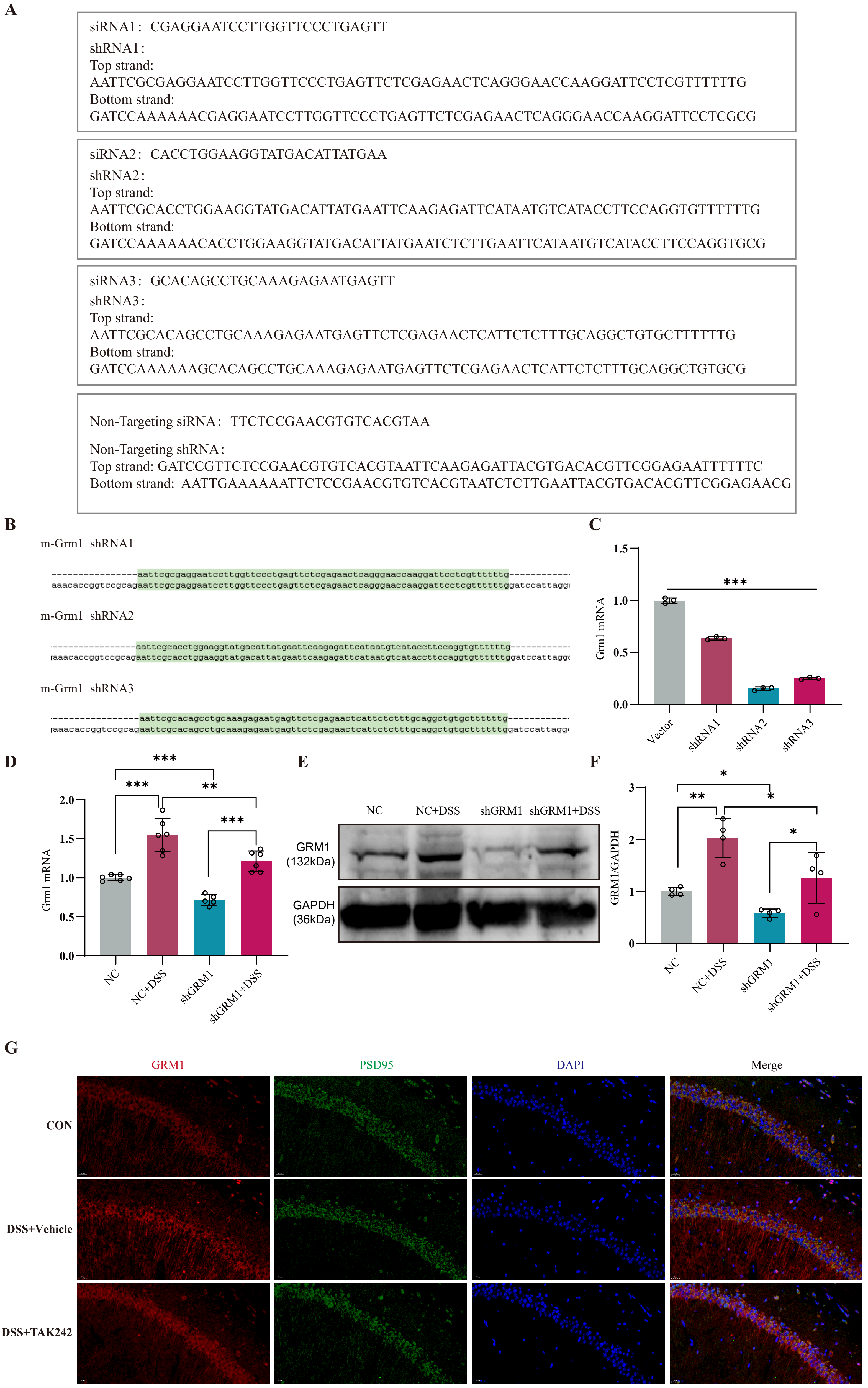
A
siRNA1: CGAGGAATCCTTGGTTCCCTGAGTT
shRNA1:
Top strand:
AATTCGCGAGGAATCCTTGGTTCCCTGAGTTCTCGAGAACTCAGGGAACCAAGGATTCCTCGTTTTTTG
Bottom strand:
GATCCAAAAAACGAGGAATCCTTGGTTCCCTGAGTTCTCGAGAACTCAGGGAACCAAGGATTCCTCGCG
siRNA2: CACCTGGAAGGTATGACATTATGAA
shRNA2:
Top strand:
AATTCGCACCTGGAAGGTATGACATTATGAATTCAAGAGATTCATAATGTCATACCTTCCAGGTGTTTTTTG
Bottom strand:
GATCCAAAAAACACCTGGAAGGTATGACATTATGAATCTCTTGAATTCATAATGTCATACCTTCCAGGTGCG
siRNA3: GCACAGCCTGCAAAGAGAATGAGTT
shRNA3:
Top strand:
AATTCGCACAGCCTGCAAAGAGAATGAGTTCTCGAGAACTCATTCTCTTTGCAGGCTGTGCTTTTTTG
Bottom strand:
GATCCAAAAAAGCACAGCCTGCAAAGAGAATGAGTTCTCGAGAACTCATTCTCTTTGCAGGCTGTGCG
Non-Targeting siRNA: TTCTCCGAACGTGTCACGTAA
Non-Targeting shRNA:
Top strand: GATCCGTTCTCCGAACGTGTCACGTAATTCAAGAGATTACGTGACACGTTCGGAGAATTTTTTC
Bottom strand: AATTGAAAAAATTCTCCGAACGTGTCACGTAATCTCTTGAATTACGTGACACGTTCGGAGAACG
B
m-Grm1 shRNA1
m-Grm1 shRNA2
m-Grm1 shRNA3
C
Grm1 mRNA
Vector
shRNA1
shRNA2
shRNA3
D
Grm1 mRNA
NC
NC+DSS
shGRM1
shGRM1+DSS
E
NC
NC+DSS
shGRM1
shGRM1+DSS
GRM1 (132kDa)
GAPDH (36kDa)
F
GRM1/GAPDH
NC
NC+DSS
shGRM1
shGRM1+DSS
G
GRM1
PSD95
DAPI
Merge
CON
DSS+Vehicle
DSS+TAK242
